# Residual risk persists in severely calcified lesions despite optimal stent expansion: a paradoxical finding from an IVUS-guided PCI cohort

**DOI:** 10.3389/fcvm.2026.1897344

**Published:** 2026-09-28

**Authors:** Shaowu Xiao, Mengya Zeng, Ying Liang, Yingying Mo, Yuewu Chen

**Affiliations:** 1 Second Clinical College, Hainan Medical University, Haikou, China; 2 Department of Cardiovascular Internal Medicine, The Second Affiliated Hospital of Hainan Medical University, Haikou, China

**Keywords:** coronary artery calcification, intravascular ultrasound, paradoxical risk, percutaneous coronary intervention, stent expansion, target vessel revascularization

## Abstract

**Background:**

Severe coronary artery calcification (CAC) is associated with adverse events after percutaneous coronary intervention (PCI), traditionally attributed to inadequate stent expansion. Whether severe CAC confers residual risk despite optimal acute mechanical results remains unknown.

**Methods:**

This single-center retrospective study included 709 patients who underwent pre-procedural intravascular ultrasound (IVUS) before PCI. Patients were classified as no calcification (*n* = 417), mild CAC (arc ≤180° or length ≤5 mm, *n* = 168), or severe CAC (arc >180° and length >5 mm, *n* = 124). The primary endpoint was clinically driven target vessel revascularization (TVR); the secondary endpoint was major adverse cardiovascular events (MACE). Post-PCI minimum stent area (MSA) and expansion index were compared across groups. Multivariable Cox regression using three prespecified adjustment models was performed.

**Results:**

Over a median follow-up of 18.6 months, 57 patients (8.0%) experienced TVR and 122 (17.2%) experienced MACE. Post-PCI MSA (5.9 vs. 5.6 vs. 5.8 mm^2^; *P* = 0.286) and expansion index (0.89 vs. 0.88 vs. 0.88; *P* = 0.485) were comparable across groups, indicating acceptable acute stent expansion in severely calcified lesions. Nevertheless, severe CAC remained independently associated with increased TVR risk in the primary analysis (adjusted hazard ratio [aHR] 2.51, 95% CI 1.33–4.74, *P* = 0.005), persisting after extended adjustment (aHR 2.61, *P* = 0.007) and after excluding rotational atherectomy (RA) cases (aHR 2.29, *P* = 0.018). The association between severe CAC and MACE was more modest (aHR 1.77, *P* = 0.014) and attenuated after extended adjustment (aHR 1.49, *P* = 0.119). Calcified nodules, present in 64.5% of severe CAC lesions, independently predicted TVR (aHR 3.04, *P* < 0.001). Mild CAC showed no significant risk elevation.

**Conclusion:**

Severe CAC identifies a subgroup at persistently elevated risk for repeat revascularization despite achieving stent expansion comparable to non-calcified lesions, challenging the conventional paradigm linking calcification to adverse events solely through underexpansion.

## Central Illustration

**Figure:**
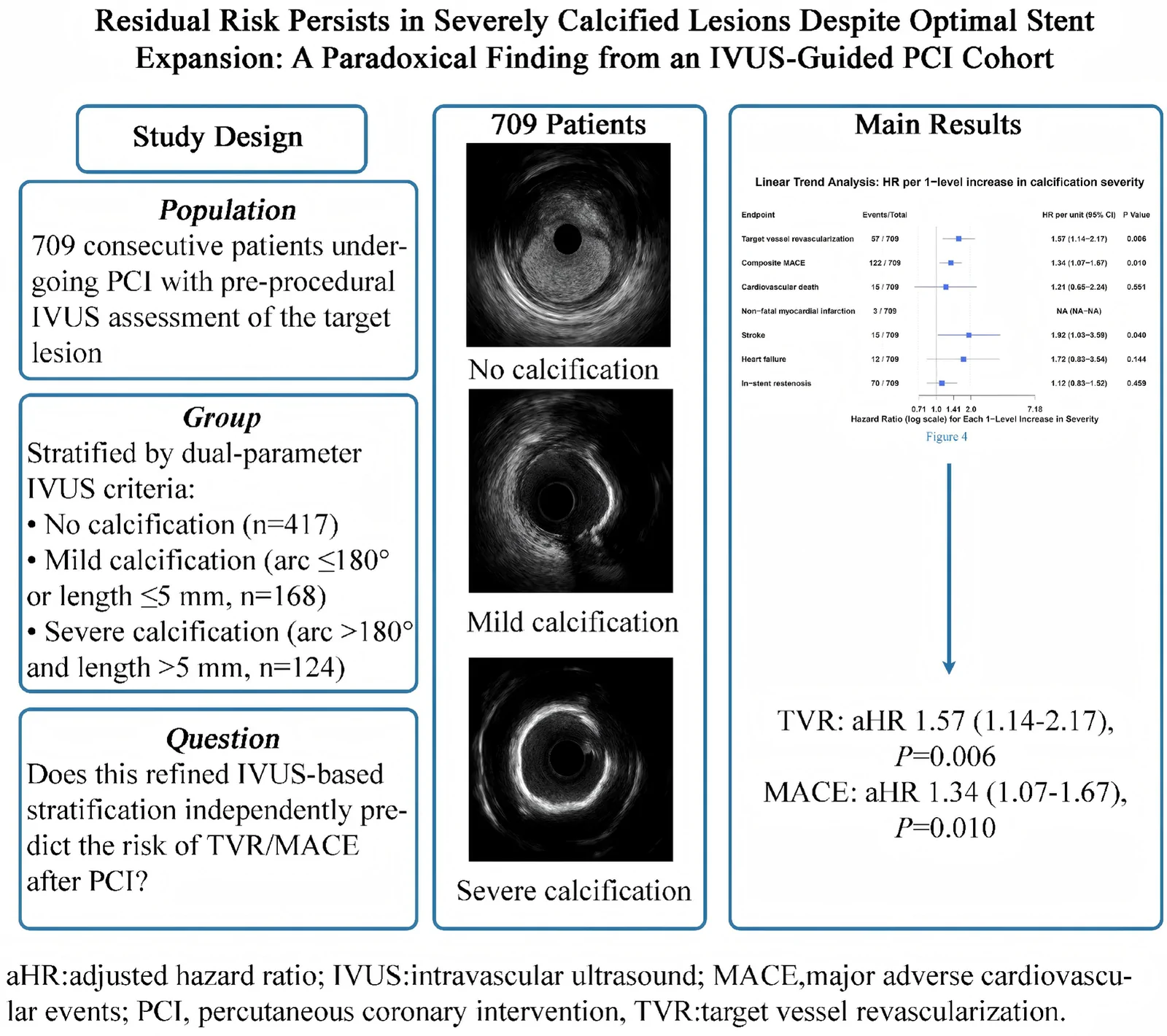

## 1 Background

Coronary artery calcification (CAC) is a hallmark of advanced atherosclerosis and a well-recognized challenge during percutaneous coronary intervention (PCI). Severe calcification impairs stent delivery, limits optimal expansion, and predisposes to in-stent restenosis (ISR) and stent thrombosis (1, 2). Intravascular ultrasound (IVUS) provides high-resolution cross-sectional imaging that detects calcium with far greater sensitivity than angiography (3) and guides lesion preparation and stent optimization.

Current expert consensus recommends integrating both calcium arc and longitudinal length for procedural planning (4). A dual-parameter classification—mild (arc ≤180° or length ≤5 mm) versus severe (arc >180° and length >5 mm)—has been proposed to identify lesions warranting dedicated calcium modification strategies (4). This classification has been validated for predicting stent underexpansion in selected cohorts (5). The prognostic value of calcium quantification has also been extended to noninvasive imaging. A recent study by Yang et al. demonstrated that lesion-specific CAC score >200 derived from computed tomography was associated with an approximately 25-fold increased risk of stent underexpansion, underscoring the potential of pre-procedural calcium scoring for risk stratification prior to invasive angiography (6).

The traditional mechanistic paradigm posits that severe CAC leads to adverse clinical outcomes primarily through inadequate stent expansion. Consequently, optimal lesion preparation and IVUS-guided stent deployment are expected to mitigate this risk by achieving acceptable minimum stent area (MSA) and expansion indices. However, whether severe CAC confers residual risk of adverse events even after optimal acute stent expansion has been achieved remains unknown. This question is clinically critical: if aggressive lesion preparation and IVUS optimization eliminate the excess risk, then current strategies are sufficient; if a residual risk persists, novel therapeutic approaches are needed.

To date, no study has directly examined whether IVUS-optimized stent expansion mitigates the excess risk associated with severe calcification. In this study, we tested the paradoxical hypothesis that severe CAC—defined by IVUS as arc >180° and length >5 mm—independently predicts target vessel revascularization (TVR) and major adverse cardiovascular events (MACE) despite achieving post-PCI stent expansion comparable to that in non-calcified lesions. We aimed to: 1) validate the dual-parameter classification in a real-world IVUS-guided PCI cohort; 2) compare post-PCI MSA and expansion indices across calcification severity groups; and 3) determine whether severe CAC confers residual risk after accounting for acute mechanical results and extended clinical covariates.

## 2 Methods

### 2.1 Study design and population

This single-center retrospective cohort study was approved by the Ethics Committee of The Second Affiliated Hospital of Hainan Medical University (Approval No. LW2022035) and conducted in accordance with the Declaration of Helsinki. The requirement for written informed consent was waived due to the retrospective use of deidentified data.

Consecutive patients who underwent coronary angiography and preprocedural IVUS between January 1, 2023, and July 31, 2024, were screened. Of 762 initially identified records, 53 were excluded due to poor image quality (*n* = 2), missing identifiers (*n* = 9), or duplicate admissions (*n* = 42). The final analysis cohort comprised 709 patients (Figure 1).

**Figure 1:**
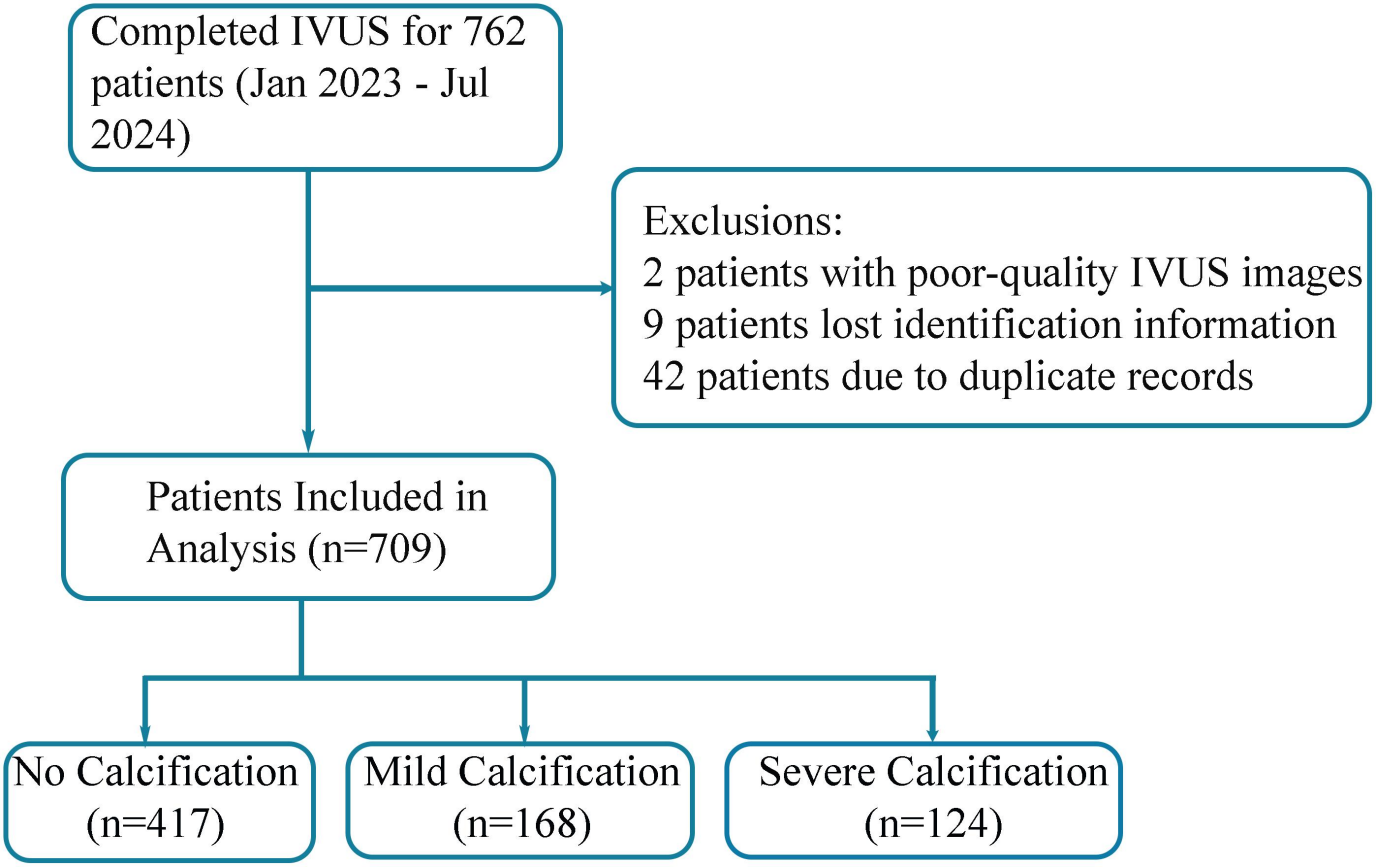
Patient screening and study cohort flow diagram. IVUS, intravascular ultrasound.

Inclusion criteria: 1) age ≥18 years; 2) coronary angiography with pre-procedural IVUS performed between January 1, 2023, and July 31, 2024; 3) PCI performed on the IVUS-assessed vessel. Patients with chronic total occlusion (CTO; *n* = 30) and ISR (*n* = 96) were included only if PCI was successful (final TIMI 3 flow and residual diameter stenosis <30%).

Exclusion criteria: 1) poor IVUS image quality precluding reliable calcium assessment; 2) missing patient identifiers preventing follow-up; 3) duplicate admission records; 4) failed CTO recanalization; 5) failed ISR intervention; 6) severe valvular heart disease requiring surgical or transcatheter intervention during the index hospitalization; 7) malignant tumors with expected survival <1 year; and 8) end-stage renal disease with eGFR <15 mL/min/1.73 m^2^ not on maintenance dialysis. Patients on maintenance dialysis were included.

All enrolled patients were followed from the date of the index PCI until the predefined study termination date of September 30, 2025. Follow-up was completed in all patients, with no losses to follow-up across the three CAC severity groups. As the last patient was enrolled on July 31, 2024, the minimum potential follow-up duration for any participant was at least 14 months, with a median follow-up of 18.6 months (IQR 15.5–23.2 months).

### 2.2 IVUS acquisition and CAC classification

Preintervention IVUS was performed using an iLab™ Polaris Multi-Modality Guidance System (Boston Scientific) with automated pullback at 1 mm/s. Offline analysis was conducted independently by two experienced interventional cardiologists blinded to clinical outcomes. Calcification was defined as a hyperechoic lesion with acoustic shadowing (7). In cases of ISR, calcium within the neointima and peri-stent regions was assessed using identical criteria. Calcified nodules were identified as convex, protruding calcific masses with irregular surfaces and acoustic shadowing, as previously described (8).

Inter-observer agreement. Inter-observer agreement was assessed in all 709 patients. For calcium arc (categorized as 0°, 1–90°, 91–180°, 181–270°, >270°), the intraclass correlation coefficient (ICC) using a two-way mixed-effects model for absolute agreement was 0.981 (95% CI: 0.978–0.984). For calcium length (continuous), the ICC was 0.999 (95% CI: 0.999–0.999). For calcified nodule (binary), Cohen's kappa was 0.930 (95% CI: 0.896–0.965). For severity group (three-level categorical), Cohen's kappa was 0.955 (95% CI: 0.935–0.975). All metrics indicated “almost perfect” agreement per Landis and Koch criteria. Discrepancies were resolved by consensus conference with a third senior interventional cardiologist (Y.C.) serving as adjudicator.

For each target lesion, the maximum calcium arc (degrees) and longitudinal calcium length (mm) were measured. Patients were stratified into three mutually exclusive groups based on *a priori* dual-parameter criteria:

1. No calcification: No detectable calcium.
2. Mild calcification: Any calcium not meeting severe criteria (arc ≤180° or length ≤5 mm).
3. Severe calcification: Arc >180° and length >5 mm (4).

Representative IVUS images of the three categories are shown in Figure 2.

**Figure 2:**
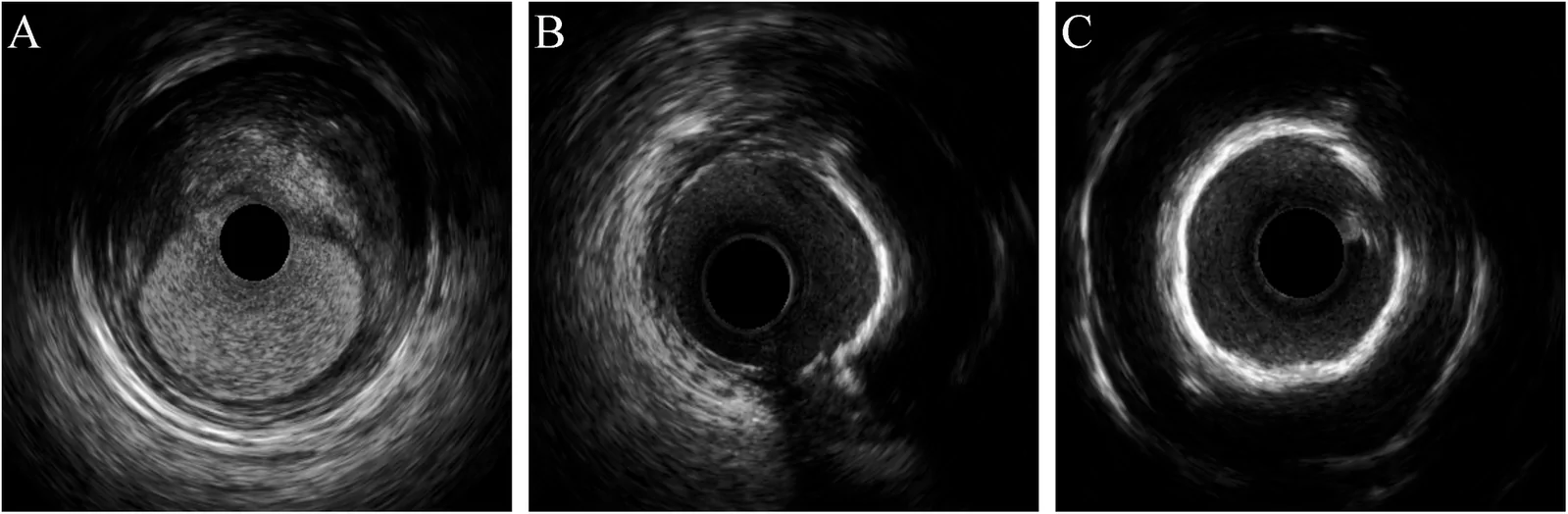
Representative intravascular ultrasound (IVUS) images showing different degrees of calcification. **(A)** No calcification. **(B)** Mild calcification. **(C)** Severe calcification. Figures from authors’ organization.

Unit of analysis. The unit of analysis was the patient. For each patient, the culprit lesion was defined as the lesion responsible for the clinical presentation (in acute coronary syndrome [ACS]) or the most functionally significant lesion (in stable angina, determined by fractional flow reserve when available, or by angiographic severity ≥70% or 50%–70% with documented ischemia). In patients with ISR, the ISR lesion was considered the culprit lesion if it was responsible for the clinical presentation. In patients who underwent IVUS of more than one vessel (*n* = 112, 15.8%), only the culprit lesion was used for CAC severity classification.

### 2.3 Post-PCI IVUS analysis and stent expansion metrics

Post-PCI IVUS was performed at the discretion of the treating operator based on the following criteria: 1) suboptimal angiographic result (residual stenosis ≥20% or haziness at the stent edge); 2) complex lesions (left main, bifurcation, long lesions, small vessels, severe calcification); 3) clinical concern for stent thrombosis or dissection; or 4) routine quality assurance. The following metrics were measured offline by investigators blinded to clinical outcomes:

1. MSA: The smallest luminal cross-sectional area within the stented segment (mm^2^).
2. Stent expansion index: MSA divided by the predicted stent area based on nominal stent diameter (calculated as π × [nominal radius]^2^) (9).

### 2.4 Data collection and endpoints

Demographic, clinical, laboratory, and procedural data were extracted from electronic medical records. Stent types used were exclusively second-generation drug-eluting stents (DES).

Lesion preparation strategies included cutting/scoring balloons and high-pressure non-compliant balloons (≥20 atm). In all patients treated with rotational atherectomy (RA; *n* = 24), cutting/scoring balloons and high-pressure non-compliant balloon post-dilatation were routinely performed per institutional protocol. Ultra-high-pressure balloons (≥30 atm) were not available at our center during the study period.

Procedural success was defined as achievement of final TIMI 3 flow with residual angiographic diameter stenosis <30%, in the absence of in-hospital major adverse events. Stent optimization under IVUS guidance adhered to standard criteria, including stent expansion index >0.80, complete stent apposition, and absence of significant edge dissection (defined as medial involvement >60° or length >2 mm) (4). Given the inclusion of small-diameter stents (including 2.25 mm stents), the criterion of MSA >5.5 mm^2^ was not applied.

Endpoints. The primary endpoint was clinically driven TVR, defined as repeat revascularization of the target vessel—specifically the vessel containing the culprit lesion used for CAC classification—due to angiographic diameter stenosis ≥50% with documented ischemia (symptoms or objective evidence) or stenosis ≥70% regardless of symptoms. Revascularization of non-target vessels due to disease progression was not counted as TVR.

The secondary endpoint was the composite of MACE, defined as cardiovascular death, nonfatal myocardial infarction, ischemic stroke, clinically driven TVR, hospitalization for heart failure, or angiographically confirmed ISR. All events were adjudicated by an independent Clinical Events Committee using prespecified definitions (10, 11).

### 2.5 Statistical analysis

Categorical variables are presented as counts (percentages) and continuous variables as median (IQR). Group comparisons used the Kruskal–Wallis test or *χ*^2^ test (or Fisher's exact test) as appropriate.

A key pre-specified analysis was the comparison of post-PCI IVUS metrics (MSA and expansion index) across CAC groups using the Kruskal–Wallis test. This analysis was designed to challenge the traditional mechanistic assumption that severe CAC inevitably leads to inferior stent expansion.

Cumulative event rates were estimated using the Kaplan–Meier method with 95% confidence intervals and compared with the log-rank test. Multivariable Cox proportional hazards models were employed to assess the independent association between CAC severity and outcomes.

Three prespecified adjustment models:

1. Model A (primary): age, sex, smoking, hypertension, dyslipidemia, diabetes mellitus, eGFR.
2. Model B (extended): Model A plus log-transformed NT-proBNP, log-transformed hs-CRP, LVEF, stent diameter, multiple stents, and RA. Calcified nodule was not included in Model B due to near-collinearity with severe CAC (Cramér's V = 0.63; 64.5% of severe CAC lesions harbored calcified nodules vs. 0% of non-calcified lesions); its effect was assessed separately.
3. Model C (parsimonious extended): Model A plus log-transformed NT-proBNP, log-transformed hs-CRP, and RA.

CAC severity was modeled both as an ordinal variable (no calcification=0, mild=1, severe=2) and as a categorical variable with dummy coding (reference = no calcification).

Pre-specified sensitivity and subgroup analyses: 1) single-parameter continuous Cox models for arc and length in the calcified subgroup (Supplementary Table S1); 2) stratification by ACS (STEMI + NSTEMI + unstable angina) versus stable angina; 3) exclusion of patients with baseline ISR; 4) exclusion of patients treated with RA; and 5) complete-case analysis.

All tests were two-sided, and *P* < 0.05 was considered statistically significant. Analyses were performed using R software version 4.5.2.

## 3 Results

### 3.1 Baseline and procedural characteristics

Among 709 patients, 417 (58.8%) had no calcification, 168 (23.7%) had mild calcification, and 124 (17.5%) had severe calcification. Patients with severe CAC were older, had a higher prevalence of hypertension and diabetes, lower eGFR, and higher lipoprotein(a) levels compared with the other groups (all *P* < 0.05; Table 1). Clinical presentation (ACS vs. non-ACS) did not differ significantly across groups (*P* = 0.212).

**Table 1:** Baseline characteristics according to coronary artery calcification severity.

	Overall	No calcification	Mild calcification	Severe calcification	*P*
	(*n* = 709)	*n* =417 (58.8%)	*n* = 168 (23.7%)	*n*= 124 (17.5%)	value
Patient demographics					
Age, years	65 (58, 71)	64 (57, 69)	66 (59, 72)	68 (61, 75)	<0.001
Gender, *n* (%)					0.279
Male	543 (76.6)	328 (78.7)	125 (74.4)	90 (72.6)	
Female	166 (23.4)	89 (21.3)	43 (25.6)	34 (27.4)	
Body mass index, kg/m^2^	23.8 (21.6, 25.7)	23.9 (21.6, 25.7)	23.7 (21.6, 25.9)	23.5 (21.9, 25.7)	0.967
SBP, mmHg	135 (121,154)	135 (122, 153)	135 (120, 155)	136 (119, 156)	0.905
DBP, mmHg	79 (71,89)	80 (71, 90)	78 (71, 88)	78 (71, 88)	0.455
Medical history, *n* (%)					
Smoking	207 (29.2)	127 (30.5)	53 (31.6)	27 (21.8)	0.130
Hypertension	420 (59.2)	229 (54.9)	104 (61.9)	87 (70.2)	0.007
Dyslipidemia	136 (19.2)	81 (19.4)	34 (20.2)	21 (16.9)	0.763
Diabetes mellitus	240 (33.9)	127 (30.5)	61 (36.3)	52 (41.9)	0.045
Prior myocardial infarction	134 (18.9)	76 (18.2)	36 (21.4)	22 (17.7)	0.627
Prior PCI	222 (31.3)	132 (31.7)	47 (28.0)	43 (34.7)	0.462
Laboratory					
uric acid, μmol/L	364 (301,436)	360 (297,432)	361 (296,430)	380 (319,449)	0.154
Hemoglobin A1c, %	6.3 (5.9,7.1)	6.2 (5.8,6.9)	6.3 (6,7.2)	6.3 (5.9,7.5)	0.034
eGFR, mL/min/1.73 m^2^	89.0 (72.0,101.0)	92.3 (76.0,104.0)	84.7 (70.0,97.7)	82.0 (60.5,94.0)	<0.001
Triglycerides, mmol/L	1.37 (0.96,1.94)	1.37 (0.97,1.92)	1.36 (0.97,2.05)	1.31 (0.94,1.97)	0.946
Total cholesterol, mmol/L	4.63 (3.7,5.47)	4.65 (3.71,5.49)	4.71 (3.85,5.52)	4.20 (3.43,5.35)	0.055
LDL-C, mmol/L	2.84 (2.15,3.58)	2.88 (2.21,3.61)	2.90 (2.31,3.68)	2.51 (1.93,3.37)	0.004
HDL-C, mmol/L	1.06 (0.9,1.24)	1.08 (0.93,1.26)	1.02 (0.85,1.24)	1.02 (0.86,1.19)	0.030
Lipoprotein (a), mg/dL	11.10 (4.20,28.83)	8.20 (3.63,24.07)	16.09 (6.12,36.59)	13.69 (6.46,31.20)	<0.001
CK-MB, U/L	13 (10,24)	13 (10,24)	14 (9,23)	14 (9,24)	0.895
Cardiac troponin I, ng/mL	0.01 (0.01,0.99)	0.01 (0.01,0.82)	0.01 (0.01,1.32)	0.01 (0.01,1.4)	0.222
NT-proBNP, pg/mL	207 (64,963)	144 (52,685)	312 (92,1292)	338 (102,1728)	<0.001
hs-CRP, mg/L	3.14 (1.14,8.67)	3.00 (1.12,6.25)	4.14 (1.38,13.65)	3.92 (1.06,10.61)	0.015
LVEF, %	62 (54,66)	62 (56,67)	61 (54,65)	59 (48,64)	<0.001
Clinical presentation, *n* (%)					0.212
STEMI	106 (15.0)	66 (15.8)	26 (15.5)	14 (11.3)	0.450
NSTEMI	113 (15.9)	59 (14.2)	29 (17.3)	25 (20.2)	0.239
Unstable angina	367 (51.8)	225 (54.0)	86 (51.2)	56 (45.2)	0.224
Stable angina	123 (17.3)	67 (16.1)	27 (16.1)	29 (23.4)	0.148
Comorbidities, *n* (%)					
Aortic valve calcification	44 (6.2)	14 (3.4)	17 (10.1)	13 (10.5)	0.001
NAVC	20 (2.8)	6 (1.4)	4 (2.4)	10 (8.1)	0.001
Aortic Stenosis	5 (0.7)	1 (0.2)	1 (0.6)	3 (2.4)	0.038
Discharge Medications, *n* (%)					
ACEI/ARB	417 (58.8)	231 (55.4)	108 (64.3)	78 (62.9)	0.084
Beta-blocker	473 (66.7)	267 (64.0)	112 (66.7)	94 (75.8)	0.051
statins	693 (97.7)	408 (97.8)	163 (97.0)	122 (98.4)	0.724
Dual antiplatelet therapy	643 (90.7)	375 (89.9)	157 (93.5)	111 (89.5)	0.367
Aspirin	659 (92.9)	384 (92.1)	162 (96.4)	113 (91.1)	0.122
Clopidogrel	390 (55.0)	215 (51.6)	98 (58.3)	77 (62.1)	0.072
Ticagrelor	301 (42.5)	189 (45.3)	66 (39.3)	46 (37.1)	0.170

Data are presented as median (interquartile range) for continuous variables and number (%) for categorical variables. *P* values were calculated using the Kruskal‒Wallis test for continuous variables and the *χ*^2^ test or Fisher's exact test for categorical variables, as appropriate.

ACE-I/ARBs, angiotensin-converting enzyme inhibitors/angiotensin receptor blockers; CK-MB, creatine kinase myocardial band; DBP, diastolic blood pressure; eGFR, glomerular filtration rate; HDL-C, high-density lipoprotein; hs-CRP, high-sensitivity C-reactive protein; LDL-C, low-density lipoprotein; LVEF, left ventricular ejection fraction; NAVC, Nonaortic valve calcification; NSTEMI, non-ST segment elevation acute myocardial infarction; NT-proBNP, N-terminal pro-B type natriuretic peptide; PCI, percutaneous coronary intervention; SBP, systolic blood pressure; STEMI, ST segment elevation acute myocardial infarction.

Stent implantation was more frequent in the mild (89.9%) and severe (84.7%) CAC groups than in the no-CAC group (76.7%, *P* = 0.001). Multiple stents were used more often in calcified lesions (*P* < 0.001). Calcified nodules were identified in 131 patients (18.5%) overall, with a pronounced gradient across groups: 0% in no CAC, 30.4% in mild CAC, and 64.5% in severe CAC (*P* < 0.001; Table 2). RA was predominantly performed in the severe CAC group (16.9% vs. 0.2% in no CAC, *P* < 0.001; Table 2). Procedural success was achieved in 703 patients (99.2%) overall, with no significant difference among groups (no CAC: 412 [98.8%]; mild: 168 [100%]; severe: 123 [99.2%]; *P* = 0.358; Table 2). The overall use of cutting/scoring balloons was 6.5% (46/709), with a significant gradient across calcification severity (2.4% in no CAC, 7.7% in mild CAC, and 18.5% in severe CAC; *P* < 0.001; Table 2). High-pressure non-compliant balloons were used in 56.7% of all procedures (402/709), with higher rates in more severely calcified lesions (49.9%, 60.1%, and 75.0%, respectively; *P* < 0.001; Table 2).

**Table 2:** Procedural characteristics according to coronary artery calcification severity.

	Overall	No calcification	Mild calcification	Severe calcification	*P* value
	(*n* = 709)	*n* =417 (58.8%)	*n* = 168 (23.7%)	*n*= 124 (17.5%)	
Target vessel assessed by IVUS, *n* (%)					
LM	147 (20.7)	87 (20.9)	37 (22.0)	23 (18.5)	0.765
LAD	508 (71.7)	293 (70.3)	118 (70.2)	97 (78.2)	0.202
LCX	103 (14.5)	66 (15.8)	26 (15.5)	11 (8.9)	0.143
RCA	162 (22.8)	93 (22.3)	44 (26.2)	25 (20.2)	0.440
Lesion characteristics, n(%)					
In-stent restenosis	96 (13.5)	56 (13.4)	21 (12.5)	19 (15.3)	0.780
Calcified nodule	131 (18.5)	0	51 (30.4)	80 (64.5)	<0.001
Chronic total occlusion	30 (4.2)	24 (5.8)	2 (1.2)	4 (3.2)	0.038
Bifurcation	30 (4.2)	19 (4.6)	8 (4.8)	3 (2.4)	0.541
Procedural details, n(%)					
Treated with stent implantation	576 (81.2)	320 (76.7)	151 (89.9)	105 (84.7)	0.001
Single stent	225 (31.7)	150 (36.0)	46 (27.4)	29 (23.4)	0.012
Multiple stents	351 (49.5)	170 (40.8)	105 (62.5)	76 (61.3)	<0.001
Treated with DEB	96 (13.5)	60 (14.4)	17 (10.1)	19 (15.3)	0.321
Single DEB	73 (10.3)	42 (10.1)	14 (8.3)	17 (13.7)	0.319
Multiple DEB	23 (3.2)	18 (4.3)	3 (1.8)	2 (1.6)	0.156
Procedural success	703 (99.2)	412 (98.8)	168 (100.0)	123 (99.2)	0.358
Lesion preparation strategies, n(%)					
Cutting/scoring balloons	46 (6.5)	10 (2.4)	13 (7.7)	23 (18.5)	<0.001
High-pressure non-compliant balloon (≥20 atm)	402 (56.7)	208 (49.9)	101 (60.1)	93 (75.0)	<0.001
Intraprocedural adjunctive therapy, n(%)					
Intraprocedural use of GPI	130 (18.3)	83 (19.9)	30 (17.9)	17 (13.7)	0.289
Intraprocedural use of IABP	17 (2.4)	7 (1.7)	4 (2.4)	6 (4.8)	0.130
Rotational atherectomy	24 (3.4)	1 (0.2)	2 (1.2)	21 (16.9)	<0.001

Data are presented as number (percentage). *P* values were calculated using the *χ*^2^ test or Fisher's exact test, as appropriate.

DEB, drug-eluting balloon; GPI, glycoprotein IIb/IIIa inhibitor; IABP, intra-aortic balloon pump; IVUS, intravascular ultrasound; LAD, left anterior descending artery; LCX, left circumflex artery; LM, left main coronary artery; PCI, percutaneous coronary intervention; RCA, right coronary artery.

### 3.2 Post-PCI stent expansion: No difference across groups

Post-procedural IVUS was performed in 388 patients (54.7% of the cohort), with similar availability across groups (no CAC: 229/417 [54.9%]; mild: 95/168 [56.5%]; severe: 64/124 [51.6%]; *P* = 0.699). Patients who underwent post-PCI IVUS had a higher rate of multiple stenting (57.7% vs. 39.6%, *P* < 0.001; Supplementary Table S7), reflecting the selective use of post-PCI imaging in challenging cases.

Contrary to the traditional mechanistic paradigm, MSA and stent expansion indices did not differ significantly among the three groups (Table 3). Median MSA was 5.9 mm^2^ (IQR 5.0–6.9) in no CAC, 5.6 mm^2^ (4.6–6.7) in mild CAC, and 5.8 mm^2^ (4.9–6.9) in severe CAC (*P* = 0.286). Similarly, the stent expansion index was comparable across groups (median 0.89, 0.88, and 0.88, respectively; *P* = 0.485).

**Table 3:** Post-PCI intravascular ultrasound metrics by calcification severity.

	No calcification	Mild calcification	Severe calcification	*P*
	*n* =417 (58.8%)	*n* = 168 (23.7%)	*n*= 124 (17.5%)	value
Post-procedural imaging performed	229 (54.9%)	95 (56.5%)	64 (51.6%)	0.699
Minimum stent area, mm^2^	5.9 (5.0–6.9)	5.6 (4.6–6.7)	5.8 (4.9–6.9)	0.286
Stent expansion index	0.89 (0.85–0.92)	0.88 (0.84–0.93)	0.88 (0.84–0.92)	0.485

These findings indicate that under IVUS guidance, severely calcified lesions can achieve stent expansion comparable to non-calcified lesions when appropriately prepared with more aggressive lesion modification strategies, including higher rates of cutting/scoring balloons (18.5% vs. 7.7% vs. 2.4%, *P* < 0.001) and high-pressure non-compliant balloons (75.0% vs. 60.1% vs. 49.9%, *P* < 0.001; Table 2). This does not imply that severe CAC inherently behaves like non-calcified plaque; rather, the comparable final expansion reflects the deliberate application of advanced preparation techniques in calcified lesions.

### 3.3 Clinical outcomes: paradoxical persistence of TVR risk

Over a median follow-up of 18.6 months (IQR 15.5–23.2) through the censoring date of September 30, 2025, 57 patients (8.0%) experienced clinically driven TVR and 122 patients (17.2%) experienced at least one MACE.

#### 3.3.1 Primary endpoint: target vessel revascularization

Despite comparable acute stent expansion across groups, severe CAC remained associated with a significantly higher risk of clinically driven TVR in all analyses (Table 4, Figure 3). In the ordinal analysis, each one-grade increase in CAC severity conferred a 57% higher adjusted risk of TVR (aHR 1.57, 95% CI 1.14–2.17; *P* = 0.006; Figure 4). In the categorical analysis using no CAC as reference (Table 5; Figure 5), severe CAC was independently associated with a 2.5-fold increased risk of TVR in the primary model (aHR 2.51, 95% CI 1.33–4.74; *P* = 0.005).

**Table 4:** Association between coronary artery calcification severity (ordinal) and clinical outcomes.

Event, *n* (%)	Overall	No calcification	Mild calcification	Severe calcification	aHR (95% CI)	P for trend
	(*n* = 709)	*n* =417 (58.8%)	*n* = 168 (23.7%)	*n*= 124 (17.5%)		
Primary endpoint						
TVR	57 (8.0)	26 (6.2)	14 (8.3)	17 (13.7)	1.57 (1.14–2.17)	0.006
Secondary endpoints						
Composite MACE	122 (17.2)	56 (13.4)	34 (20.2)	32 (25.8)	1.34 (1.07–1.67)	0.010
Cardiovascular death	15 (2.1)	6 (1.4)	2 (1.2)	7 (5.6)	1.21 (0.65–2.24)	0.551
Non-fatal MI	3 (0.4)	0	2 (1.2)	1 (0.8)	—	—
Stroke	15 (2.1)	5 (1.2)	4 (2.4)	6 (4.8)	1.92 (1.03–3.59)	0.040
Heart failure	12 (1.7)	3 (0.7)	3 (1.8)	6 (4.8)	1.72 (0.83–3.54)	0.144
In-stent restenosis	70 (9.9)	37 (8.9)	19 (11.3)	14 (11.3)	1.12 (0.83–1.52)	0.459

Data are presented as number (percentage). Adjusted hazard ratios (aHRs) with 95% confidence intervals (CIs) are presented for a per 1-grade increase in coronary artery calcification severity (modeled as an ordinal categorical variable: no calcification=0, mild=1, severe=2). Results shown are from Model A, adjusted for age, sex, smoking, hypertension, dyslipidemia, diabetes mellitus, and estimated glomerular filtration rate. Results from extended adjustment models (Models B and C) are presented in Supplementary Table S6. The dash (—) indicates that the aHR was not estimated due to an insufficient number of events.

aHR, adjusted hazard ratio; CI, confidence interval; MACE, major adverse cardiovascular events; MI, myocardial infarction; TVR, target vessel revascularization.

**Figure 3:**
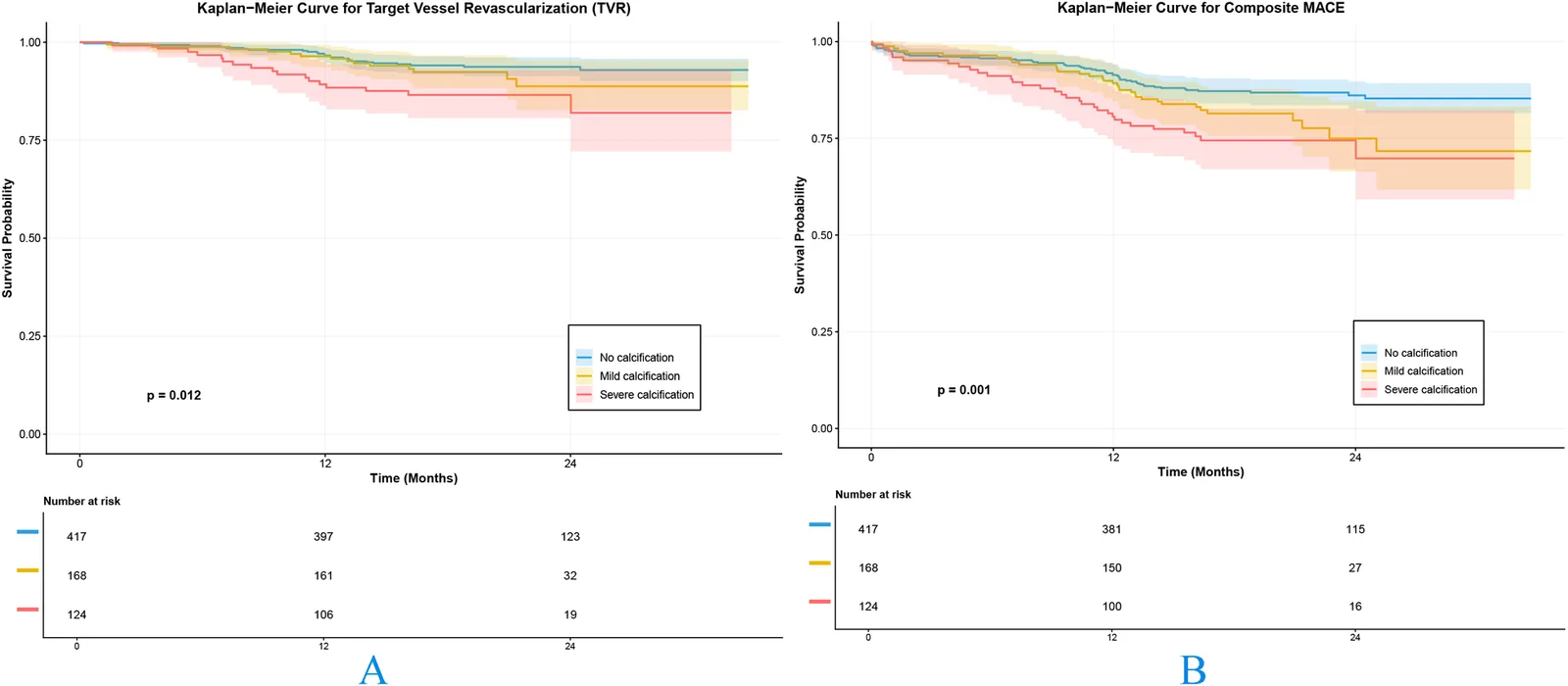
Kaplan–meier curves for the primary endpoint of target vessel revascularization (TVR) **(A)** and the secondary endpoint of major adverse cardiovascular events (MACE) **(B).**

**Figure 4:**
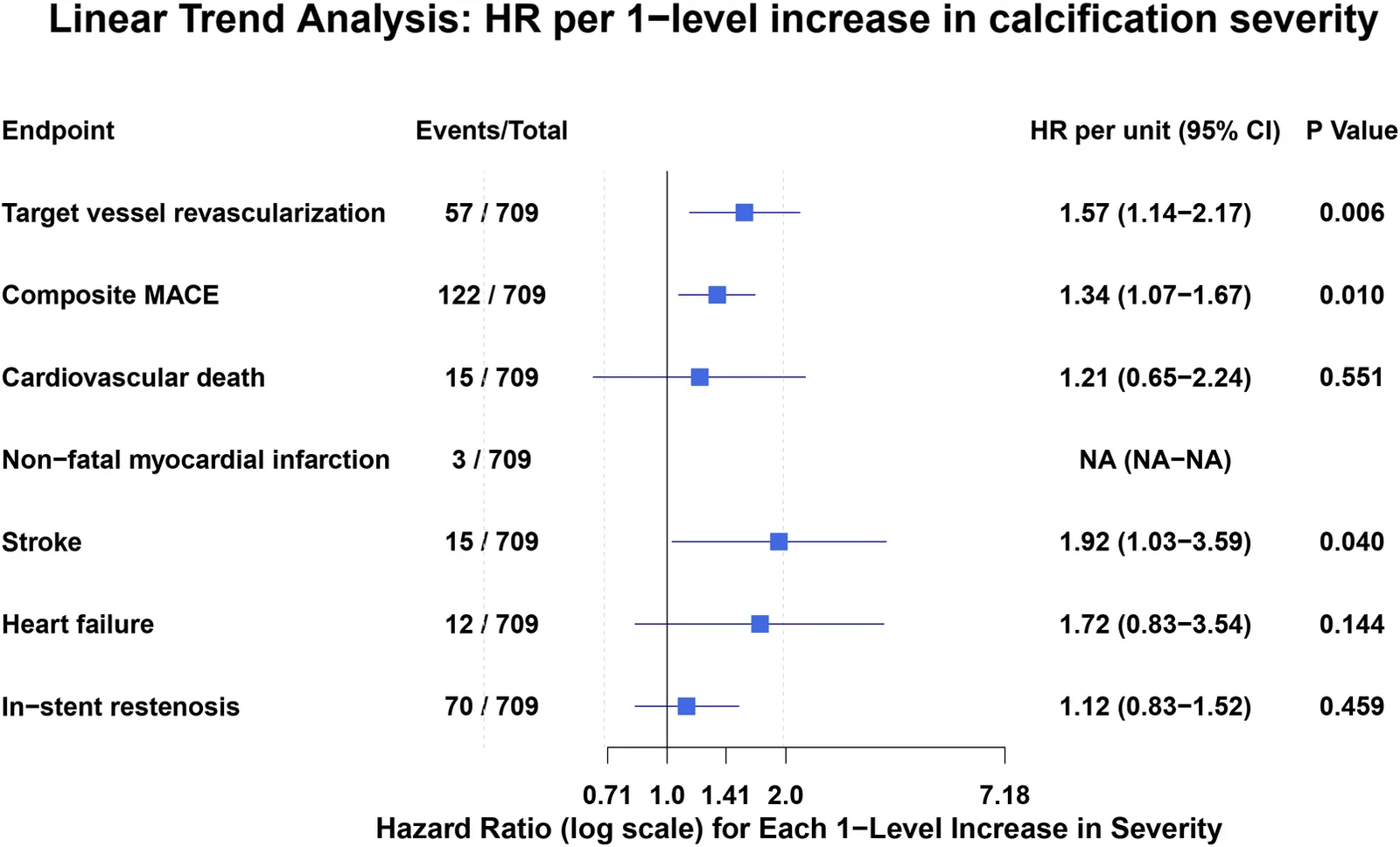
Forest plot for the ordinal association between coronary artery calcification severity and clinical outcomes. The Cox proportional hazards model was adjusted for the following covariates: age, sex, smoking, hypertension, dyslipidemia, diabetes mellitus, and estimated glomerular filtration rate. MACE, major adverse cardiovascular events; NA, the aHR was not estimated due to an insufficient number of events.

**Table 5:** Associations between coronary artery calcification severity categories and clinical outcomes.

	Comparison	aHR (95% CI)	*P* value
Primary endpoint			
TVR	Mild vs No	1.35 (0.70–2.61)	0.368
	Severe vs No	2.51 (1.33–4.74)	0.005
Secondary endpoints			
Composite MACE	Mild vs No	1.45 (0.95–2.24)	0.088
	Severe vs No	1.77 (1.12–2.78)	0.014
Cardiovascular death	Mild vs No	0.70 (0.14–3.49)	0.660
	Severe vs No	1.49 (0.45–4.89)	0.512
Non-fatal MI	Mild vs No	—	—
	Severe vs No	—	—
Stroke	Mild vs No	1.94 (0.51–7.34)	0.330
	Severe vs No	3.70 (1.06–12.95)	0.041
Heart failure	Mild vs No	2.09 (0.42–10.44)	0.367
	Severe vs No	3.00 (0.68–13.31)	0.148
In-stent restenosis	Mild vs No	1.25 (0.71–2.19)	0.433
	Severe vs No	1.22 (0.64–2.30)	0.549

The reference group is patients with no coronary artery calcification. Calcification severity was modeled as a categorical variable with dummy coding (Mild vs. No; Severe vs. No). Results shown are from Model A, adjusted for age, sex, smoking, hypertension, dyslipidemia, diabetes mellitus, and estimated glomerular filtration rate. Results from extended adjustment models (Models B and C) are presented in Supplementary Table S6. The dash (—) indicates that the aHR was not estimated due to an insufficient number of events.

aHR, adjusted hazard ratio; CI, confidence interval; MACE, major adverse cardiovascular events; MI, myocardial infarction; TVR, target vessel revascularization.

**Figure 5:**
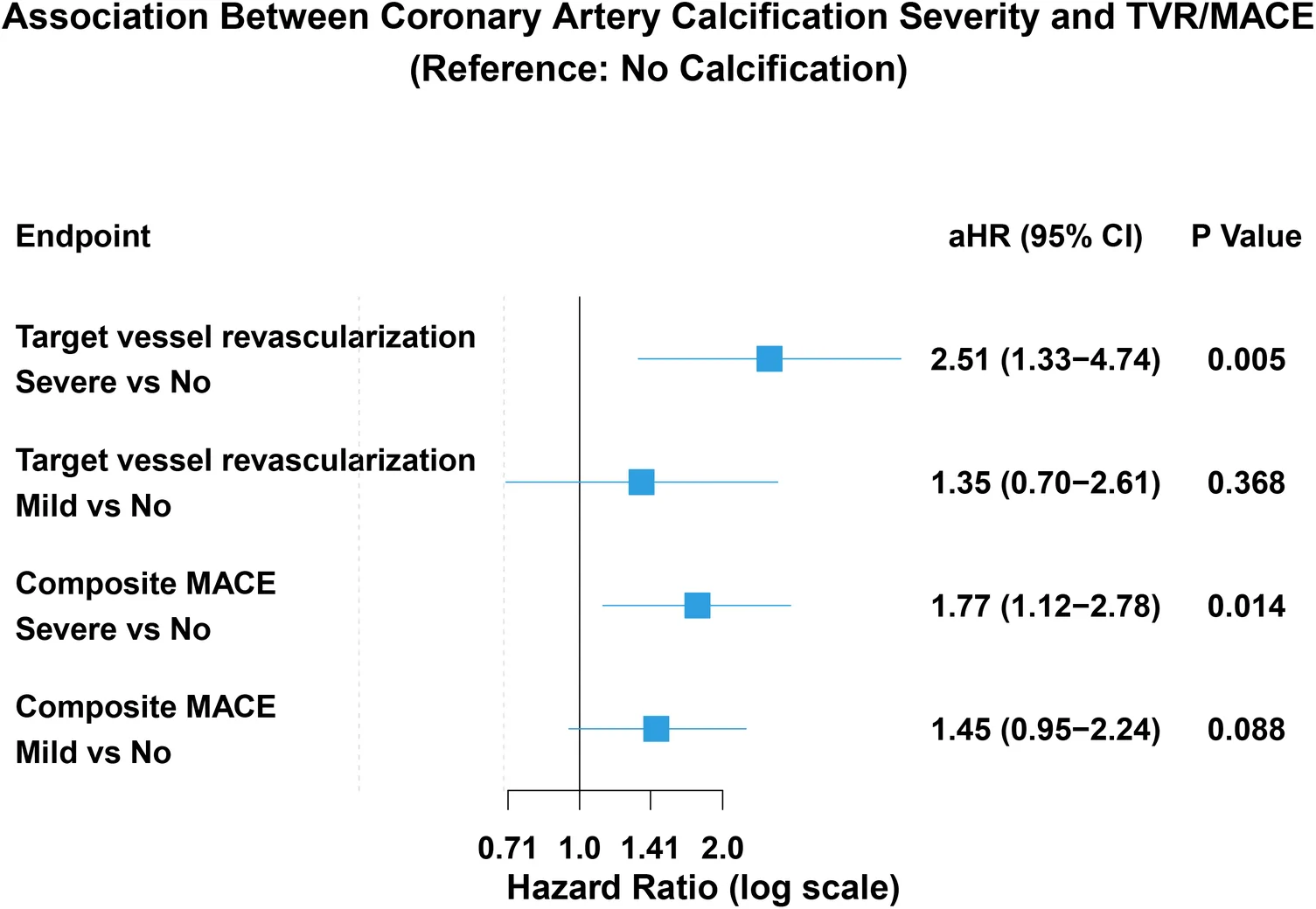
Forest plot of target vessel revascularization (TVR) and major adverse cardiovascular events (MACE) according to severity of coronary artery calcification (reference: no calcification). Adjusted for age, sex, smoking, hypertension, dyslipidemia, diabetes mellitus, and estimated glomerular filtration rate.

Robustness to extended adjustment. The association between severe CAC and TVR remained statistically significant after extended adjustment in both Model B (aHR 2.61, 95% CI 1.30–5.20; *P* = 0.007) and Model C (aHR 2.49, 95% CI 1.25–4.92; *P* = 0.009)(Supplementary Table S6), indicating that the observed excess risk was not explained by inflammatory markers, NT-proBNP, LVEF, stent characteristics, or the use of RA (Table 4). Mild CAC did not show statistically significant risk elevation for TVR in any model (Model A: aHR 1.35, 95% CI 0.70–2.61; *P* = 0.368).

#### 3.3.2 Secondary endpoint: composite MACE

Severe CAC was associated with an increased risk of composite MACE in the primary analysis (Model A: aHR 1.77, 95% CI 1.12–2.78; *P* = 0.014; Table 5). In ordinal analysis, each one-grade increase in CAC severity conferred a 34% higher adjusted risk of MACE (aHR 1.34, 95% CI 1.07–1.67; *P* = 0.010). However, this association was attenuated after extended adjustment (Model B: aHR 1.49, 95% CI 0.90–2.46; *P* = 0.119; Model C: aHR 1.54, 95% CI 0.94–2.52; *P* = 0.086), suggesting that a portion of the MACE risk may be mediated by systemic factors—including inflammation and neurohormonal activation—rather than by the local mechanical effects of the index lesion alone (Table 4). Mild CAC did not show statistically significant risk elevation for MACE in any model (Model A: aHR 1.45, 95% CI 0.95–2.24; *P* = 0.089).

#### 3.3.3 Calcified nodule: an independent predictor

Calcified nodule, present in 64.5% of severe CAC lesions but absent in non-calcified lesions, was independently associated with TVR (aHR 3.04, 95% CI 1.74–5.30; *P* < 0.001) and MACE (aHR 2.00, 95% CI 1.35–2.97; *P* = 0.001) after adjustment for age, sex, smoking, hypertension, dyslipidemia, diabetes, and eGFR.

#### 3.3.4 Individual secondary endpoints

In ordinal analysis, stroke demonstrated a significant association with CAC severity (aHR 1.92, 95% CI 1.03–3.59; *P* = 0.040), and in categorical analysis, severe CAC was associated with a 3.7-fold increased risk of stroke compared with no calcification (aHR 3.70, 95% CI 1.06–12.95; *P* = 0.041). However, this association was attenuated after extended adjustment (Model B: aHR 2.83, 95% CI 0.69–11.69; *P* = 0.150), suggesting that systemic inflammatory burden may partly mediate this relationship.

No significant associations were observed for cardiovascular death (aHR 1.49, 95% CI 0.45–4.89; *P* = 0.511), heart failure hospitalization (aHR 3.00, 95% CI 0.68–13.31; *P* = 0.148), or ISR (aHR 1.22, 95% CI 0.64–2.30; *P* = 0.549). The number of nonfatal myocardial infarctions was extremely low (*n* = 3, all in calcified groups), precluding multivariable adjustment for this endpoint. Kaplan–Meier curves for individual secondary endpoints are presented in the Supplementary Figure.

### 3.4 Sensitivity and subgroup analyses

Exclusion of ISR lesions (Supplementary Table S3). After excluding 96 patients with baseline ISR, the point estimates for severe CAC attenuated (TVR aHR 1.52, 95% CI 0.70–3.29; *P* = 0.286; MACE aHR 1.34, 95% CI 0.79–2.27; *P* = 0.277) and were no longer statistically significant. This suggests that the ISR subgroup contributed materially to the association observed in the overall cohort. Given the limited number of ISR patients (*n* = 96), this *post-hoc* analysis is hypothesis-generating only.

Exclusion of RA (Supplementary Table S4). Excluding 24 patients treated with RA did not materially alter the TVR risk associated with severe CAC (aHR 2.29, 95% CI 1.15–4.54; *P* = 0.018) or the MACE risk (aHR 1.69, 95% CI 1.04–2.76; *P* = 0.035). The persistence of elevated risk after excluding these cases suggests that the observed association is not solely driven by the use of RA but rather reflects an inherent property of severely calcified lesions.

ACS vs. stable angina stratification (Supplementary Table S2). In the ACS subgroup (*n* = 586), severe CAC was strongly associated with both TVR (aHR 3.17, 95% CI 1.53–6.54; *P* = 0.002) and MACE (aHR 2.04, 95% CI 1.25–3.34; *P* = 0.004). In the stable angina subgroup (*n* = 123), the associations were not statistically significant (TVR aHR 1.37, 95% CI 0.28–6.62; *P* = 0.699; MACE aHR 1.24, 95% CI 0.35–4.31; *P* = 0.740), likely reflecting limited statistical power. No significant interaction was observed (TVR P-interaction = 0.115; MACE P-interaction = 0.161).

Complete-case analysis (*n* = 680) yielded results nearly identical to the imputed analysis (Supplementary Table S5).

## 4 Discussion

### 4.1 Main findings

This study yields three principal findings. First, in a real-world IVUS-guided PCI cohort, a dual-parameter IVUS classification of CAC severity—defined by maximum arc >180° and longitudinal length >5 mm—effectively stratifies patients at significantly elevated risk for both TVR and MACE. Second, and most critically, this elevated risk endures despite the achievement of post-PCI MSA and stent expansion indices that are statistically and clinically comparable to those attained in non-calcified and mildly calcified lesions. Third, mild CAC does not confer a statistically significant excess risk when managed under contemporary IVUS guidance.

We emphasize that TVR represents the most mechanistically appropriate endpoint for testing the hypothesis that severe calcification confers lesion-specific residual risk. The composite MACE endpoint, while clinically relevant, includes events (stroke, heart failure hospitalization, cardiovascular death) that may be driven primarily by systemic vascular disease burden rather than by the local effects of the index lesion. The stronger and more robust association observed for TVR supports a lesion-specific mechanism, whereas the weaker and attenuated MACE association likely reflects the contribution of systemic disease burden.

The paradoxical observation of acceptable acute stent expansion yet persistently higher TVR rates fundamentally challenges the traditional mechanistic paradigm that attributes adverse outcomes in calcified lesions primarily to inadequate stent deployment. Our findings demonstrate that the relationship between severe calcification and adverse events extends beyond the issue of acute stent underexpansion, implicating more complex, long-term biological and biomechanical mechanisms.

### 4.2 Why does residual risk persist despite optimal stent expansion?

Several non-mutually exclusive mechanisms may explain this paradox.

First, chronic stent eccentricity and asymmetric geometry with resultant disturbed flow. While MSA measures the minimum cross-sectional area, it fails to capture the three-dimensional spatial distribution of stent struts relative to the vessel wall. Severely calcified plaques frequently create crescent-shaped or irregular lumens that, even after technically successful high-pressure balloon expansion, result in eccentric stent geometry. Recent advances in computational fluid dynamics have demonstrated that eccentric stent configurations generate highly disturbed local hemodynamic patterns, including oscillatory wall shear stress and regions of flow stasis (12). Low and oscillatory wall shear stress are potent pro-atherogenic and pro-proliferative stimuli that upregulate endothelial adhesion molecules, promote smooth muscle cell migration, and accelerate neointimal hyperplasia (13).

Second, edge effects at the calcific plaque–compliant vessel transition zone. The abrupt interface between a heavily calcified, non-compliant arterial segment and an adjacent non-calcified, physiologically distensible segment creates a mechanical stress concentration point. During each cardiac cycle, cyclic vessel wall motion generates repetitive hinge motion at these transition zones. Even when the stented segment itself is optimally expanded, the edges are subjected to abnormal mechanical strain, promoting edge restenosis through sustained activation of mechanosensitive signaling pathways (13).

Third, severe calcification as a marker of a distinct, biologically active disease phenotype. We propose that severe calcification—particularly when accompanied by calcified nodules—represents more than a mechanical obstacle; it identifies a distinct vascular disease phenotype characterized by chronic, smoldering inflammation and persistent calcification activity. Calcified plaques are metabolically active tissues with high expression of osteogenic differentiation markers and inflammatory cytokines (14). The mechanical injury from balloon dilatation and stent deployment in this pro-inflammatory microenvironment may trigger an exaggerated and sustained healing response, leading to accelerated neointimal hyperplasia independent of the quality of acute stent expansion. This is supported by our finding that calcified nodule was independently associated with a 3-fold increased risk of TVR (aHR 3.04, *P* < 0.001), even after adjustment for baseline covariates.

Fourth, the role of calcified nodules. Calcified nodules, identified in 64.5% of our severe CAC group, represent a particularly high-risk morphologic feature. Unlike sheet calcium, calcified nodules are characterized by protruding, irregular calcific masses that can penetrate into the lumen and disrupt the overlying fibrous cap (8). In a large OCT-based registry, calcified nodules were independently associated with a > 2-fold increased risk of target lesion failure at 2 years, driven predominantly by early stent thrombosis and late restenosis (15). A recent comprehensive review by Jinnouchi et al. reported that eruptive calcified nodules are associated with higher rates of TVR despite achieving greater stent expansion compared with non-eruptive nodules (16). This striking parallel to our primary paradoxical finding strongly suggests that the presence of calcified nodules, particularly the eruptive subtype, may be a key driver of the observed excess risk.

Fifth, the contribution of lesion preparation strategies. In our cohort, severely calcified lesions required significantly more aggressive lesion preparation, including RA (16.9% vs. 0.2%), cutting/scoring balloons (18.5% vs. 2.4%), and high-pressure non-compliant balloons (75.0% vs. 49.9%). The achievement of comparable final MSA and expansion indices therefore reflects the cumulative effect of these preparatory strategies rather than an intrinsic property of calcified plaque. Critically, excluding RA cases did not eliminate the excess TVR risk (aHR 2.29, *P* = 0.018), indicating that even aggressive calcium modification does not fully address the substrate of future events.

An integrated perspective. Taken together, we propose that the residual risk observed in severely calcified lesions arises from a convergence of persistent biomechanical stressors (eccentric geometry, edge hinge motion) operating within a biologically hostile microenvironment (chronic inflammation, active calcification, calcified nodules). While optimal stent expansion addresses the acute luminal gain, it does not neutralize these ongoing pathological processes.

### 4.3 Comparison with prior literature

Our study complements and extends the robust angiographic literature demonstrating that severe calcification predicts long-term adverse events after PCI. Bourantas et al. reported that severe angiographic calcification was independently associated with increased 3-year mortality (HR 1.33) in a patient-level pooled analysis of 6,296 individuals (17). Guedeney et al. confirmed these findings in a larger cohort of 19,833 patients, demonstrating an increased 5-year risk of patient-oriented composite endpoints (HR 1.20) (18). Rheude et al. extended these observations to 10 years (19). More recently, Sato et al. demonstrated that calcified nodules independently predicted 2-year target lesion failure (HR 2.13) (15).

However, these landmark studies could not address a fundamental clinical question: does IVUS-guided optimization eliminate the excess risk associated with severe calcification? Our study fills this critical gap by providing granular, lesion-level IVUS data demonstrating that even when acute mechanical results meet or exceed contemporary optimization standards, a significant residual risk persists. This finding challenges the common assumption in interventional practice that achieving a good MSA is a sufficient endpoint in calcified lesions.

### 4.4 Interpretation of the ISR sensitivity analysis

After excluding patients with pre-existing ISR, the association between severe CAC and outcomes attenuated and lost statistical significance. This finding suggests that the ISR subgroup contributed materially to the association observed in the overall cohort. Several non-mutually exclusive explanations exist. First, the reduction in sample size (709→613) reduces statistical power, and the loss of significance may reflect a Type II error. Second, patients with ISR represent a distinct pathophysiological entity in which the dominant substrate for repeat revascularization may be neointimal hyperplasia rather than *de novo* calcium biology (20). Third, there may be a synergistic interaction between severe CAC and ISR. Importantly, this *post-hoc* analysis is underpowered to distinguish among these explanations, and we make no claims about differential applicability to *de novo* lesions. Future studies specifically designed to evaluate the prognostic role of calcium in ISR lesions are warranted.

### 4.5 Clinical implications

For the practicing interventional cardiologist, our findings convey three important messages.

First, the dual-parameter IVUS classification (arc >180° and length >5 mm) is a simple, practical tool for pre-procedural risk stratification. We recommend routine reporting of these metrics in IVUS reports for calcified lesions. Patients meeting severe CAC criteria should be counseled about a higher likelihood of future repeat revascularization, even after angiographically and IVUS-optimized PCI.

Second, the absence of significant risk elevation in mild CAC is reassuring; standard PCI techniques under IVUS guidance appear sufficient for this group, and aggressive calcium modification strategies may not be routinely necessary.

Third, and most importantly, the persistence of TVR risk despite acceptable acute stent expansion identifies an important unmet clinical need. Novel therapeutic strategies beyond improving acute mechanical expansion are required for severely calcified lesions. These may include: 1) intravascular lithotripsy (IVL), which achieves circumferential calcium modification while potentially preserving a more favorable vascular compliance and healing profile (21, 22); 2) thinner-strut DES designed to minimize chronic mechanical stress and flow disturbance (23); 3) adjunctive pharmacotherapies targeting the inflammatory and osteogenic pathways activated in calcified arteries, such as colchicine or novel anti-inflammatory agents (24); 4) extended dual antiplatelet therapy or novel antithrombotic regimens in patients with calcified nodules, given their association with late thrombotic events.

A risk-adjusted, tailored approach to calcified lesion management—one that combines optimal acute mechanical results with surveillance strategies and targeted adjunctive therapies—represents the logical next step in improving outcomes for this high-risk patient subset.

### 4.6 Limitations

Several limitations merit careful consideration.

First, the retrospective, single-center design may introduce selection bias. The prevalence of IVUS-detected calcium in our cohort (41.2%) is lower than that reported in classic series (3, 25), potentially reflecting differences in patient demographics or referral patterns, and may limit generalizability to populations with higher calcification burden.

Second, post-PCI IVUS was performed in 54.7% of patients and at the discretion of the treating operator, introducing potential selection bias. Patients who underwent post-PCI IVUS had a higher rate of multiple stenting (57.7% vs. 39.6%, *P* < 0.001; Supplementary Table S7). Although the distribution of CAC severity was similar between those with and without post-PCI imaging (*P* = 0.699), we cannot exclude the possibility that operators were more likely to perform post-PCI IVUS and persist with optimization in severely calcified lesions. This may have biased the comparison toward the null, meaning the true difference in stent expansion between groups may be greater than observed.

Third, the median follow-up of 18.6 months is adequate for capturing early and mid-term events such as ISR and TVR but is insufficient for evaluating very late events, including neoatherosclerosis progression and very late stent thrombosis.

Fourth, the low number of hard clinical events (cardiovascular death: *n* = 15; myocardial infarction: *n* = 3; stroke: *n* = 15) precludes definitive conclusions regarding these endpoints. The composite MACE endpoint is heterogeneous and includes events with pathophysiological determinants that are only partially related to the index lesion. Our findings are most robust for the endpoint of clinically driven TVR, which is the most mechanistically relevant outcome for testing the proposed hypothesis.

Fifth, the sensitivity analysis excluding ISR lesions attenuated the results, and we do not make claims about differential applicability to *de novo* lesions.

Sixth, the lack of routine post-PCI optical coherence tomography (OCT) prevented assessment of stent strut-level malapposition, tissue prolapse, and edge dissections, which are more sensitively detected by OCT and may provide additional prognostic information beyond IVUS.

Seventh, the use of second-generation DES exclusively limits generalizability to newer device platforms. However, this homogeneity also reduces confounding by stent technology.

Eighth, our classification does not incorporate calcium thickness, which is better characterized by OCT (26), and future studies should investigate whether integrating this parameter further refines risk prediction.

Ninth, the exclusion of patients with severe valvular heart disease and malignant tumors with expected survival <1 year may limit generalizability to these populations. Patients on maintenance dialysis were included, but their small number precluded separate analysis. Failed CTO and failed ISR interventions were excluded, meaning the cohort represents a population with successful PCI only. Ultra-high-pressure balloons (≥30 atm) were not available at our center during the study period, which may limit the generalizability of our findings to centers with access to more advanced calcium modification technologies.

Tenth, despite expanded multivariable adjustment (Models B and C), residual confounding cannot be excluded. Some covariates in the extended models may lie on the causal pathway between severe CAC and outcomes, and their inclusion may represent over-adjustment.

### 4.7 Future research directions

Our findings highlight several avenues for future investigation. First, prospective studies incorporating both IVUS and OCT at baseline and serial follow-up could delineate the relative contributions of underexpansion, eccentricity, and calcified nodules to long-term events. Second, randomized trials comparing different calcium modification strategies (e.g., lithotripsy vs. RA) should include long-term clinical and imaging endpoints, rather than focusing solely on acute stent expansion. Third, studies integrating computational fluid dynamics with patient-specific post-PCI geometries could quantify the hemodynamic consequences of stent eccentricity in calcified vessels. Fourth, the role of adjunctive anti-inflammatory or pleiotropic therapies in patients with severe CAC undergoing PCI represents a promising area for clinical investigation.

## 5 Conclusion

Severe coronary calcification defined by pre-procedural IVUS (arc >180° and length >5 mm) independently predicts TVR after IVUS-guided PCI. Critically, this elevated risk persists despite comparable acute stent expansion to non-calcified lesions, challenging the conventional mechanistic paradigm linking calcification to adverse events solely through underexpansion. The association with composite MACE is more modest and attenuated after extended adjustment, reflecting the contribution of systemic disease burden. Calcified nodules, present in nearly two-thirds of severe CAC lesions, represent an important high-risk morphologic feature independently associated with adverse outcomes. Mild calcification does not confer significant excess risk under IVUS guidance. These findings identify an unmet clinical need: severely calcified lesions remain a high-risk subset even after acceptable PCI, warranting investigation of novel therapeutic strategies beyond improving acute stent expansion.

## Data Availability

The original contributions presented in the study are included in the article/Supplementary Material, further inquiries can be directed to the corresponding author.

## References

[1] Ng P Maehara A Kirtane AJ McEntegart M Jaffer FA Doshi D. Management of coronary stent underexpansion. J Am Coll Cardiol. (2025) 85:625–44. 10.1016/j.jacc.2024.12.009 39939043

[2] Onnis C Virmani R Kawai K Nardi V Lerman A Cademartiri F. Coronary artery calcification: current concepts and clinical implications. Circulation. (2024) 149:251–66. 10.1161/CIRCULATIONAHA.123.065657 38227718 PMC10794033

[3] Mintz GS Popma JJ Pichard AD Kent KM Satler LF Chuang YC. Patterns of calcification in coronary artery disease. A statistical analysis of intravascular ultrasound and coronary angiography in 1155 lesions. Circulation. (1995) 91:1959–65. 10.1161/01.cir.91.7.1959 7895353

[4] Räber L Mintz GS Koskinas KC Johnson TW Holm NR Onuma Y. Clinical use of intracoronary imaging. Part 1: guidance and optimization of coronary interventions. An expert consensus document of the European association of percutaneous cardiovascular interventions. Eur Heart J. (2018) 39:3281–300. 10.1093/eurheartj/ehy285 29790954

[5] Zhang M Matsumura M Usui E Noguchi M Fujimura T Fall KN. Intravascular ultrasound-derived calcium score to predict stent expansion in severely calcified lesions. Circ Cardiovasc Interv. (2021) 14:e010296. 10.1161/CIRCINTERVENTIONS.120.010296 34665658

[6] Yang W Xu K Fu X Zhang W Hao Z Sang Z. Lesion-specific coronary artery calcium score to predict stent underexpansion. Front Cardiovasc Med. (2025) 12:1524390. 10.3389/fcvm.2025.1524390 39968339 PMC11832659

[7] Pu J Mintz GS Biro S Lee J-B Sum ST Madden SP. Insights into echo-attenuated plaques, echolucent plaques, and plaques with spotty calcification: novel findings from comparisons among intravascular ultrasound, near-infrared spectroscopy, and pathological histology in 2,294 human coronary artery segments. J Am Coll Cardiol. (2014) 63:2220–33. 10.1016/j.jacc.2014.02.576 24681142

[8] Nozoe M Nishioka S Oi K Suematsu N Kubota T. Effects of patient background and treatment strategy on clinical outcomes after coronary intervention for calcified nodule lesions. Circ Rep. (2021) 3:699–706. 10.1253/circrep.CR-21-0129 34950795 PMC8651471

[9] Lee JM Choi KH Song YB Lee J-Y Lee S-J Lee SY. Intravascular imaging-guided or angiography-guided complex PCI. N Engl J Med. (2023) 388:1668–79. 10.1056/NEJMoa2216607 36876735

[10] Thygesen K Alpert JS Jaffe AS Chaitman BR Bax JJ Morrow DA. Fourth universal definition of myocardial infarction (2018). Circulation. (2018) 138:e618–51. 10.1161/CIR.0000000000000617 30571511

[11] Metra M Tomasoni D Adamo M Bayes-Genis A Filippatos G Abdelhamid M. Worsening of chronic heart failure: definition, epidemiology, management and prevention. A clinical consensus statement by the Heart Failure Association of the European Society of Cardiology. Eur J Heart Fail. (2023) 25:776–91. 10.1002/ejhf.2874 37208936

[12] Chatzizisis YS Coskun AU Jonas M Edelman ER Feldman CL Stone PH. Role of endothelial shear stress in the natural history of coronary atherosclerosis and vascular remodeling: molecular, cellular, and vascular behavior. J Am Coll Cardiol. (2007) 49:2379–93. 10.1016/j.jacc.2007.02.059 17599600

[13] Wentzel JJ Chatzizisis YS Gijsen FJH Giannoglou GD Feldman CL Stone PH. Endothelial shear stress in the evolution of coronary atherosclerotic plaque and vascular remodelling: current understanding and remaining questions. Cardiovasc Res. (2012) 96:234–43. 10.1093/cvr/cvs217 22752349

[14] Durham AL Speer MY Scatena M Giachelli CM Shanahan CM. Role of smooth muscle cells in vascular calcification: implications in atherosclerosis and arterial stiffness. Cardiovasc Res. (2018) 114:590–600. 10.1093/cvr/cvy010 29514202 PMC5852633

[15] Sato T Matsumura M Yamamoto K Shlofmitz E Moses JW Khalique OK. Impact of eruptive vs noneruptive calcified nodule morphology on acute and long-term outcomes after stenting. JACC Cardiovasc Interv. (2023) 16:1024–35. 10.1016/j.jcin.2023.03.009 37164599

[16] Jinnouchi H Sakakura K Fujita H. Deep dive into intravascular coronary imaging in calcified lesions. Cardiovasc Interv Ther. (2025) 40:234–44. 10.1007/s12928-025-01096-8 39899261

[17] Bourantas CV Zhang Y-J Garg S Iqbal J Valgimigli M Windecker S. Prognostic implications of coronary calcification in patients with obstructive coronary artery disease treated by percutaneous coronary intervention: a patient-level pooled analysis of 7 contemporary stent trials. Heart. (2014) 100:1158–64. 10.1136/heartjnl-2013-305180 24846971

[18] Guedeney P Claessen BE Mehran R Mintz GS Liu M Sorrentino S. Coronary calcification and long-term outcomes according to drug-eluting stent generation. JACC Cardiovasc Interv. (2020) 13:1417–28. 10.1016/j.jcin.2020.03.053 32553329

[19] Rheude T Koch T Joner M Lenz T Xhepa E Wiebe J. Ten-year clinical outcomes of drug-eluting stents with different polymer coating strategies by degree of coronary calcification: a pooled analysis of the ISAR-TEST 4 and 5 randomised trials. EuroIntervention. (2023) 18:1188–96. 10.4244/EIJ-D-22-00781 36453826 PMC9936252

[20] Byrne RA Joner M Kastrati A. Stent thrombosis and restenosis: what have we learned and where are we going? The andreas grüntzig lecture ESC 2014. Eur Heart J. (2015) 36:3320–31. 10.1093/eurheartj/ehv511 26417060 PMC4677274

[21] Kereiakes DJ Di Mario C Riley RF Fajadet J Shlofmitz RA Saito S. Intravascular lithotripsy for treatment of calcified coronary lesions: patient-level pooled analysis of the disrupt CAD studies. JACC Cardiovasc Interv. (2021) 14:1337–48. 10.1016/j.jcin.2021.04.015 33939604

[22] Hill JM Kereiakes DJ Shlofmitz RA Klein AJ Riley RF Price MJ. Intravascular lithotripsy for treatment of severely calcified coronary artery disease. J Am Coll Cardiol. (2020) 76:2635–46. 10.1016/j.jacc.2020.09.603 33069849

[23] Bangalore S Toklu B Patel N Feit F Stone GW. Newer-generation ultrathin strut drug-eluting stents versus older second-generation thicker strut drug-eluting stents for coronary artery disease. Circulation. (2018) 138:2216–26. 10.1161/CIRCULATIONAHA.118.034456 29945934

[24] Nidorf SM Fiolet ATL Mosterd A Eikelboom JW Schut A Opstal TSJ. Colchicine in patients with chronic coronary disease. N Engl J Med. (2020) 383:1838–47. 10.1056/NEJMoa2021372 32865380

[25] Wang X Matsumura M Mintz GS Lee T Zhang W Cao Y. *In Vivo* calcium detection by comparing optical coherence tomography, intravascular ultrasound, and angiography. JACC Cardiovasc Imaging. (2017) 10:869–79. 10.1016/j.jcmg.2017.05.014 28797408

[26] Fujino A Mintz GS Matsumura M Lee T Kim S-Y Hoshino M. A new optical coherence tomography-based calcium scoring system to predict stent underexpansion. EuroIntervention. (2018) 13:e2182–9. 10.4244/EIJ-D-17-00962 29400655

