# Supplementary material for: Residual risk persists in severely calcified lesions despite optimal stent expansion: a paradoxical finding from an IVUS-guided PCI cohort
Source: Front Cardiovasc Med. 2026 Sep 28;13:1897344. doi: 10.3389/fcvm.2026.1897344 (PMC13654714; doi:10.3389/fcvm.2026.1897344)
Supplement: Supplementary file 1 [file Datasheet1.pdf]

Table S1. Adjusted Hazard Ratios from Single-Parameter Continuous Variable Cox Models for Calcification Arc Angle and Length in Patients with Calcification (n=292)

Endpoint	Variable	HR (95% CI)	P value
Composite MACE	Angle (per 90°)	1.19 (0.87-1.63)	0.273
	Length (per 1 mm)	0.99 (0.97-1.01)	0.535
Cardiovascular death	Angle (per 90°)	1.37 (0.55-3.41)	0.504
	Length (per 1 mm)	1.00 (0.95-1.04)	0.921
Stroke	Angle (per 90°)	1.06 (0.46-2.45)	0.883
	Length (per 1 mm)	1.01 (0.96-1.07)	0.678
TVR	Angle (per 90°)	1.47 (0.93-2.34)	0.102
	Length (per 1 mm)	0.99 (0.96-1.02)	0.659
Heart failure	Angle (per 90°)	2.04 (0.69-6.05)	0.198
	Length (per 1 mm)	0.97 (0.92-1.03)	0.348
ISR	Angle (per 90°)	0.95 (0.61-1.47)	0.806
	Length (per 1 mm)	1.01 (0.98-1.04)	0.567

Note: Models were adjusted for age, sex, smoking, hypertension, dyslipidemia, diabetes mellitus, and estimated glomerular filtration rate.

Table S2. Association Between Severe Calcification and Endpoint Events Stratified by ACS vs. Stable Angina

Endpoint	Subgroup	n(totals)	n (events)	aHR (95% CI)	P value	P for interaction
Composite MACE	ACS	586	103	2.04 (1.25–3.34)	0.004	0.161
	Stable angina	123	19	1.24 (0.35–4.31)	0.74	
TVR	ACS	586	46	3.17 (1.53–6.54)	0.002	0.115
	Stable angina	123	11	1.37 (0.28-6.62)	0.699	

Note: The non-calcification group served as the reference category for all comparisons.

Table S3. Association Between Severe Calcification and Endpoint Events After Excluding Patients with Pre-Existing ISR (n=613)

Endpoint		Original aHR (95% CI)	P value (Original)	Sensitivity aHR (95% CI)	P value (sensitivity)
Composite MACE	Mild vs No	1.45 (0.95-2.24)	0.088	1.46 (0.92-2.31)	0.109
	Severe vs No	1.77 (1.12-2.78)	0.014	1.34 (0.79-2.27)	0.277
Cardiovascular death	Mild vs No	0.70 (0.14-3.49)	0.660	0.70 (0.13-3.73)	0.675
	Severe vs No	1.49 (0.45-4.89)	0.511	1.76 (0.50-6.17)	0.379
Stroke	Mild vs No	1.94 (0.51-7.34)	0.330	1.85 (0.41-8.43)	0.427
	Severe vs No	3.70 (1.06-12.95)	0.041	3.67 (0.84-15.96)	0.083
TVR	Mild vs No	1.35 (0.70-2.61)	0.368	1.37 (0.69-2.73)	0.367
	Severe vs No	2.51 (1.33-4.74)	0.005	1.52 (0.70-3.29)	0.286
Heart failure	Mild vs No	2.09 (0.42-10.44)	0.367	1.46 (0.24-8.86)	0.683
	Severe vs No	3.00 (0.68-13.31)	0.148	2.25 (0.44-11.51)	0.332
ISR	Mild vs No	1.25 (0.71-2.19)	0.432	1.39 (0.77-2.50)	0.276
	Severe vs No	1.22 (0.64-2.30)	0.549	0.57 (0.23-1.41)	0.226

Note: After exclusion of patients with pre-existing ISR at enrollment, n = 613 remained for analysis.

Table S4. Association Between Calcification Severity and Endpoint Events After Excluding Patients Treated with Rotational Atherectomy (n=685)

Endpoint		aHR (95% CI)	P value
Composite MACE	Mild vs No	1.45 (0.94-2.24)	0.095
	Severe vs No	1.69 (1.04-2.76)	0.035
Cardiovascular death	Mild vs No	0.71 (0.14-3.56)	0.677
	Severe vs No	1.38 (0.37-5.10)	0.627
Stroke	Mild vs No	2.05 (0.54-7.78)	0.291
	Severe vs No	2.88 (0.72-11.54)	0.135
TVR	Mild vs No	1.36 (0.70-2.62)	0.363
	Severe vs No	2.29 (1.15-4.54)	0.018
Heart failure	Mild vs No	2.05 (0.41-10.24)	0.380
	Severe vs No	3.25 (0.71-14.92)	0.129
ISR	Mild vs No	1.22 (0.69-2.17)	0.489
	Severe vs No	1.26 (0.64-2.47)	0.503

Note: RA cases: n = 24; after exclusion, n = 685.

Table S5. Endpoint Events by Calcification Severity: Imputed Data (n=709) vs. Complete Cases (n=680)

Endpoint		Imputed data aHR (95% CI)	Complete case aHR (95% CI)	P value (complete case)
Composite MACE	Mild vs No	1.45 (0.95-2.24)	1.45 (0.95-2.24)	0.088
	Severe vs No	1.77 (1.12-2.78)	1.77 (1.12-2.78)	0.014
Cardiovascular death	Mild vs No	0.70 (0.14-3.49)	0.70 (0.14-3.49)	0.660
	Severe vs No	1.49 (0.45-4.89)	1.49 (0.45-4.89)	0.511
Stroke	Mild vs No	1.94 (0.51-7.34)	1.94 (0.51-7.34)	0.330
	Severe vs No	3.70 (1.06-12.95)	3.70 (1.06-12.95)	0.041
TVR	Mild vs No	1.35 (0.70-2.61)	1.35 (0.70-2.61)	0.368
	Severe vs No	2.51 (1.33-4.74)	2.51 (1.33-4.74)	0.005
Heart failure	Mild vs No	2.09 (0.42-10.44)	2.09 (0.42-10.44)	0.367
	Severe vs No	3.00 (0.68-13.31)	3.00 (0.68-13.31)	0.148
ISR	Mild vs No	1.25 (0.71-2.19)	1.25 (0.71-2.19)	0.432
	Severe vs No	1.22 (0.64-2.30)	1.22 (0.64-2.30)	0.549

Table S6. Three-Model Comparison for TVR, MACE, and Stroke (Severe vs. No Calcification)

Endpoint	Model	Severe vs No aHR (95% CI)	P value
TVR	A	2.51 (1.33–4.74)	0.005
TVR	B	2.61 (1.30–5.20)	0.007
TVR	C	2.49 (1.25–4.92)	0.009
MACE	A	1.77 (1.12–2.78)	0.014
MACE	B	1.49 (0.90–2.46)	0.119
MACE	C	1.54 (0.94–2.52)	0.086
Stroke	A	3.70 (1.06–12.95)	0.041
Stroke	B	2.83 (0.69–11.69)	0.150
Stroke	C	2.79 (0.72–10.83)	0.139

Note: Severe calcification was compared with no calcification as the reference category using categorical (dummy) modeling. Model A: adjusted for age, sex, smoking, hypertension, dyslipidemia, diabetes mellitus, and estimated glomerular filtration rate. Model B: Model A plus log-transformed NT-proBNP, log-transformed hs-CRP, LVEF, stent diameter, multiple stents, and rotational atherectomy. Model C: Model A plus log-transformed NT-proBNP, log-transformed hs-CRP, and rotational atherectomy. Calcified nodule was not included in Models B and C due to near-collinearity with severe CAC (Cramér's V = 0.63).

Abbreviations: aHR, adjusted hazard ratio; CI, confidence interval; MACE, major adverse cardiovascular events; TVR, target vessel revascularization.

Table S7. Comparison of Patients With vs. Without Post-PCI IVUS

Variable	Post-PCI IVUS (n=388)	No Post-PCI IVUS (n=321)	P value
Age, years	65 (57, 70)	66 (59, 71)	0.160
Male, n (%)	313 (80.7%)	230 (71.7%)	0.006
Diabetes, n (%)	140 (36.1%)	100 (31.2%)	0.193
eGFR, mL/min/1.73m^2	87.2 (70.0, 101.0)	90.0 (73.4, 102.0)	0.241
Severe CAC, n (%)	64 (16.5%)	60 (18.7%)	0.505
Calcified nodule, n (%)	65 (16.8%)	66 (20.6%)	0.229
ISR, n (%)	64 (16.5%)	32 (10.0%)	0.016
Rotational atherectomy, n (%)	11 (2.8%)	13 (4.0%)	0.495
Multiple stents, n (%)	224 (57.7%)	127 (39.6%)	<0.001

Note: CAC, coronary artery calcification; eGFR, estimated glomerular filtration rate; ISR, in-stent restenosis.
