# Supplementary material for: Residual risk persists in severely calcified lesions despite optimal stent expansion: a paradoxical finding from an IVUS-guided PCI cohort
Source: Front Cardiovasc Med. 2026 Sep 28;13:1897344. doi: 10.3389/fcvm.2026.1897344 (PMC13654714; doi:10.3389/fcvm.2026.1897344)
Supplement: Supplementary file 2 [file Datasheet2.pdf]

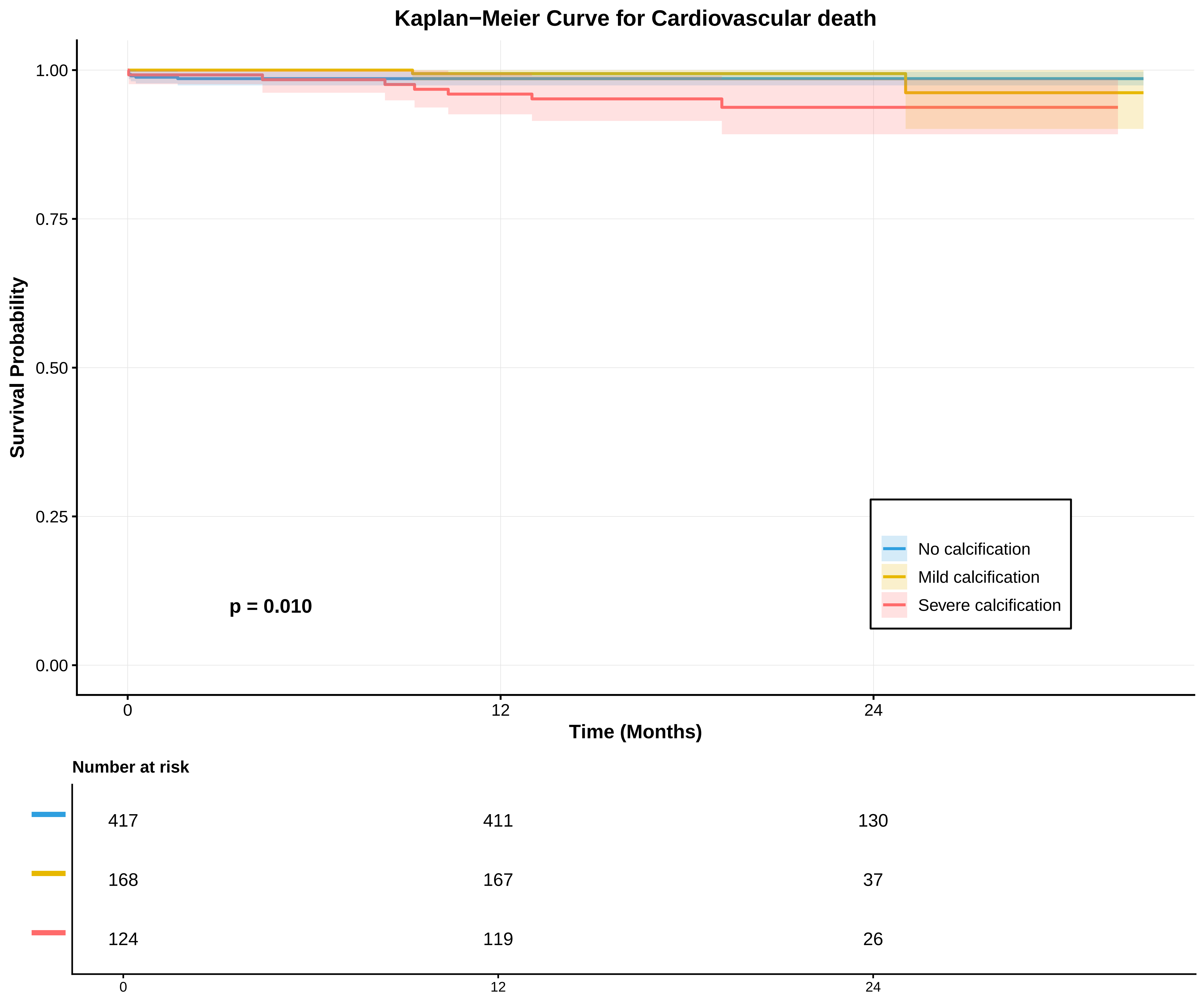
Kaplan−Meier Curve for Cardiovascular death
Survival Probability
1.00
0.75
0.50
0.25
0.00
p = 0.010
No calcification
Mild calcification
Severe calcification
0
12
24
Time (Months)
Number at risk
417
411
130
168
167
37
124
119
26
0
12
24

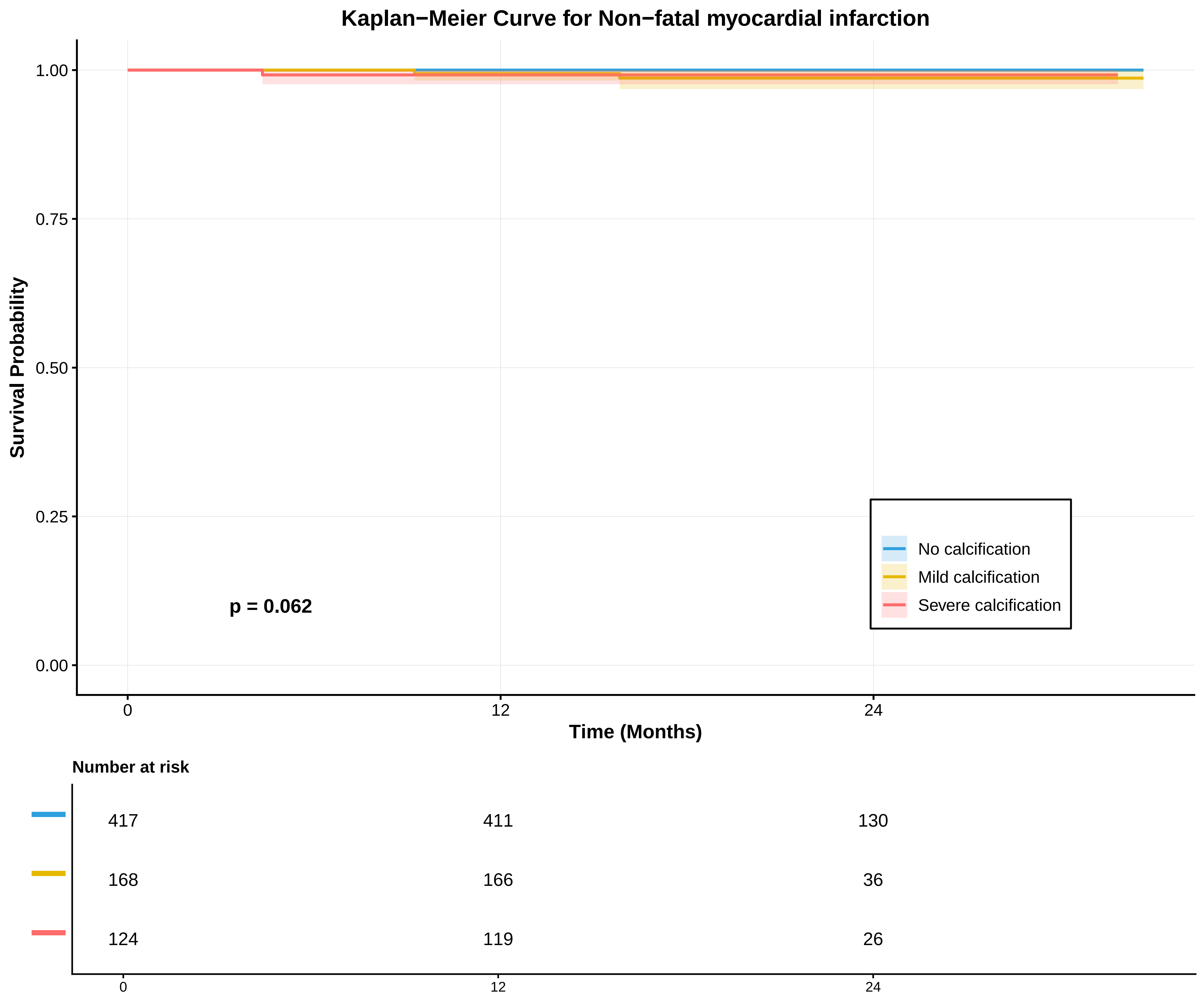
Kaplan−Meier Curve for Non−fatal myocardial infarction
Survival Probability
1.00
0.75
0.50
0.25
0.00
p = 0.062
No calcification
Mild calcification
Severe calcification
0
12
24
Time (Months)
Number at risk
417
411
130
168
166
36
124
119
26

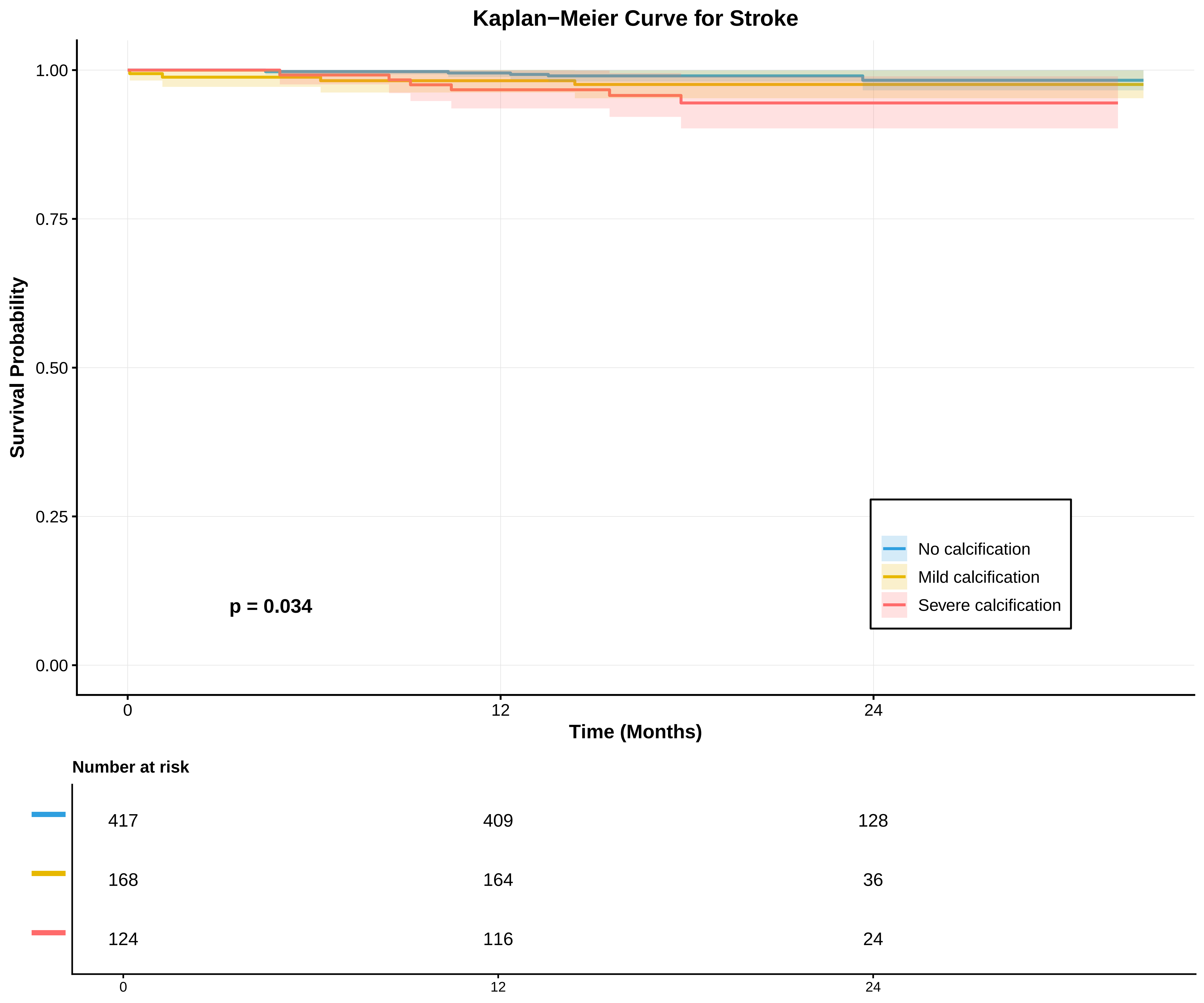
Kaplan−Meier Curve for Stroke
Survival Probability
1.00
0.75
0.50
0.25
0.00
p = 0.034
No calcification
Mild calcification
Severe calcification
0
12
24
Time (Months)
Number at risk
417
409
128
168
164
36
124
116
24
0
12
24

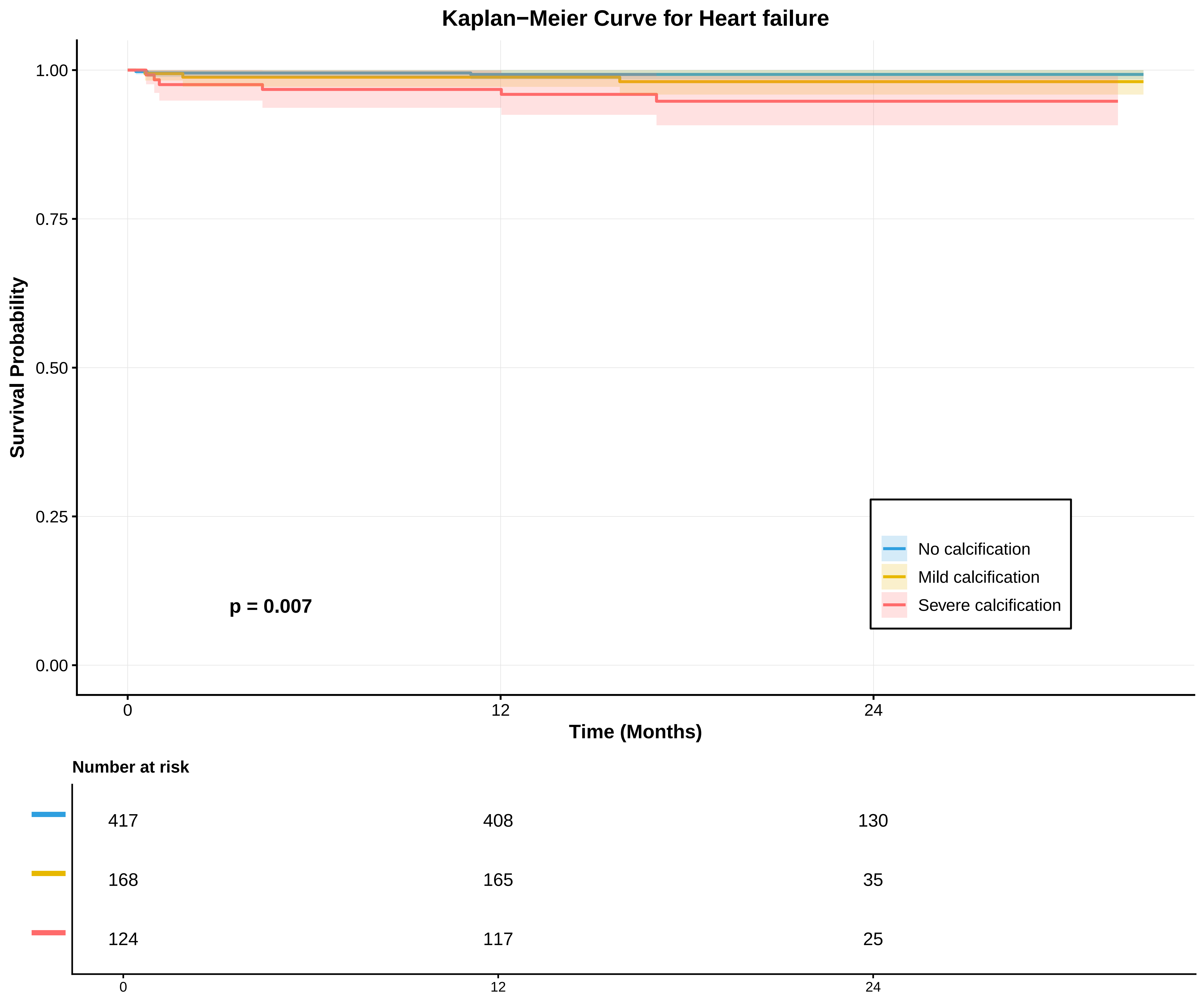
Kaplan−Meier Curve for Heart failure
Survival Probability
1.00
0.75
0.50
0.25
0.00
p = 0.007
No calcification
Mild calcification
Severe calcification
0
12
24
Time (Months)
Number at risk
417
408
130
168
165
35
124
117
25
0
12
24

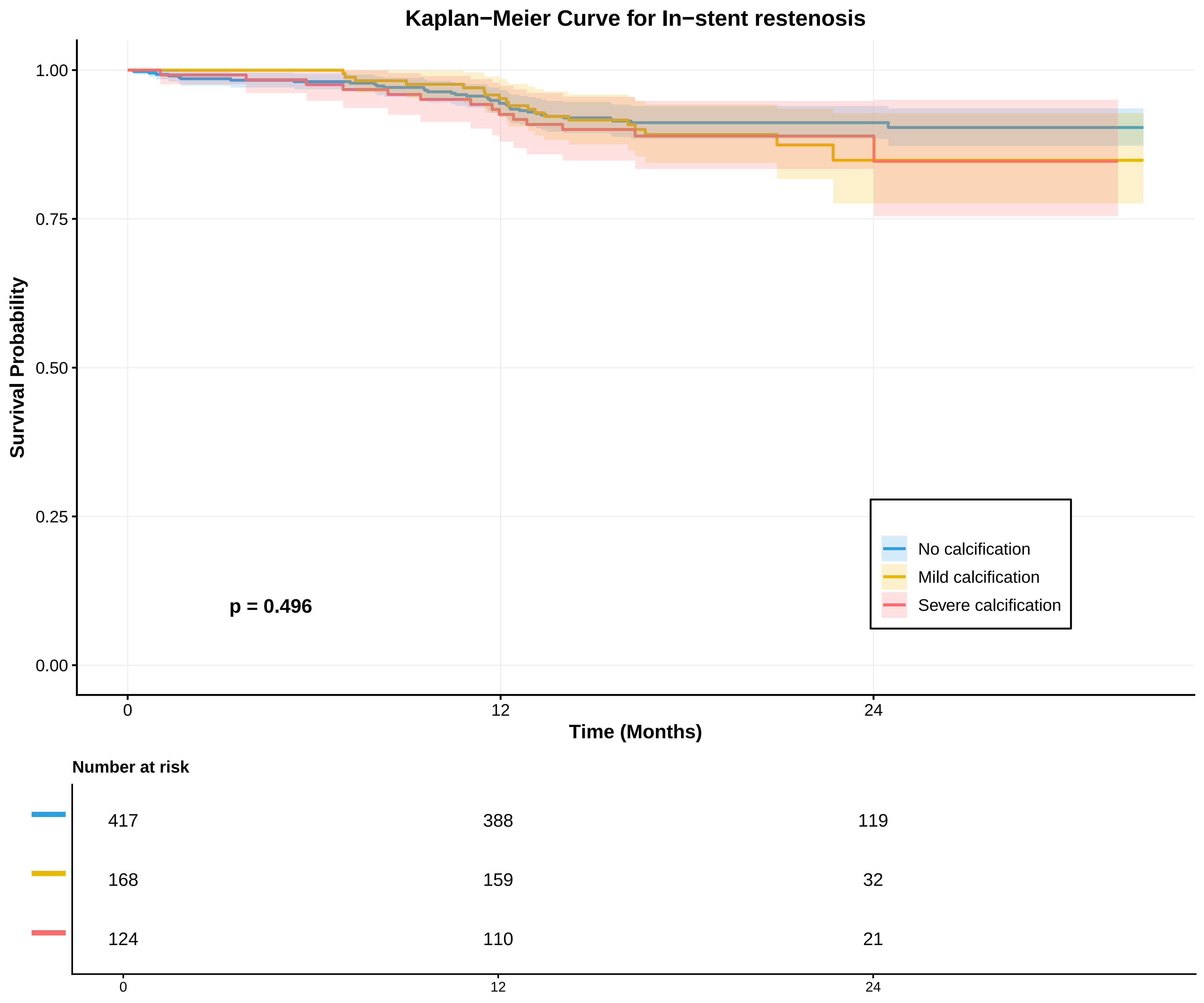
Kaplan−Meier Curve for In−stent restenosis
Survival Probability
1.00
0.75
0.50
0.25
0.00
p = 0.496
No calcification
Mild calcification
Severe calcification
0
12
24
Time (Months)
Number at risk
417
388
119
168
159
32
124
110
21
0
12
24
